# CAR T cells redirected to cell surface GRP78 display robust anti-acute myeloid leukemia activity and do not target hematopoietic progenitor cells

**DOI:** 10.1038/s41467-022-28243-6

**Published:** 2022-01-31

**Authors:** Nikhil Hebbar, Rebecca Epperly, Abishek Vaidya, Unmesha Thanekar, Sarah E. Moore, Masayuki Umeda, Jing Ma, Sagar L. Patil, Deanna Langfitt, Sujuan Huang, Cheng Cheng, Jeffery M. Klco, Stephen Gottschalk, M. Paulina Velasquez

**Affiliations:** 1 Bone Marrow Transplantation and Cellular Therapy, St. Jude Children’s Research Hospital, 262 Danny Thomas Place, Memphis, TN 38105 USA; 2 Department of Oncology, St. Jude Children’s Research Hospital, 262 Danny Thomas Place, Memphis, TN 38105 USA; 3 Department of Pathology, St. Jude Children’s Research Hospital, 262 Danny Thomas Place, Memphis, TN 38105 USA; 4 Department of Biostatistics, St. Jude Children’s Research Hospital, 262 Danny Thomas Place, Memphis, TN 38105 USA

**Keywords:** Immunotherapy, Cancer immunotherapy, Haematological cancer

## Abstract

Developing CAR T cells for acute myeloid leukemia (AML) has been hampered by a paucity of targets that are expressed on AML blasts and not on hematopoietic progenitor cells (HPCs). Here we demonstrate that GRP78 is expressed on the cell surface of primary AML blasts but not HPCs. To target GRP78, we generate T cell expressing a GRP78-specific peptide-based CAR, which show evidence of minimal fratricide post activation/transduction and antigen-dependent T cell differentiation. GRP78-CAR T cells recognize and kill GRP78-positive AML cells without toxicity to HPCs. In vivo, GRP78-CAR T cells have significant anti-AML activity. To prevent antigen-dependent T cell differentiation, we block CAR signaling and GRP78 cell surface expression post activation by using dasatinib during GRP78-CAR T cell manufacturing. This significantly improves their effector function in vitro and in vivo. Thus, targeting cell surface GRP78-positive AML with CAR T cells is feasible, and warrants further active exploration.

## Abstract (web summary)

There is an unmet need to discover suitable targets for CAR-T therapy in patients with acute myeloid leukemia (AML). Here the authors show that GRP78, a key regulator of the unfolded protein response, is highly expressed on the surface of primary AML blasts, but not on normal lymphocytes and hematopoietic progenitor cells, and that GRP78-CAR T have anti-AML activity in preclinical models.

## Introduction

Immunotherapy with T cells expressing chimeric antigen receptors (CARs) has resulted in remarkable improvement of overall survival for patients with recurrent/refractory cancers. Nevertheless, the success of CAR T cell therapy has been largely limited to B-cell lineage hematological malignancies^1–8^. P Finding an ideal immunotherapy target for AML has proven challenging due to the overlapping expression of antigens on AML blasts and normal hematopoietic progenitor cells (HPCs) or mature myeloid cells. CAR T cells have been generated against AML target antigens^9–13^. However, due to the expression of the majority of these targets (e.g., CD33, CD123) on normal hematopoietic cells, AML-redirected CAR T cells may only serve as a bridge to hematopoietic cell transplant (HCT).

While elegant strategies have been developed to reduce the risk of ‘on target/off target’ toxicity of AML-redirected CAR T cells including deleting CD33 in HPCs^14^, there is a continued need to discover antigens that are expressed solely on AML blasts and not on HPCs or mature cells of the myeloid lineage. We reason that glucose-regulated-protein 78 (GRP78, HSPA5), a key regulator on the unfolded protein response (UPR) that is evolutionarily conserved across species^15^, is a promising target for AML-redirected CAR T cell therapy since it normally resides in the endoplasmic reticulum (ER)^16^. GRP78 contains *a C-terminal KDEL sequence that acts as retention sequence by binding its receptor in the ER (KDEL-R1)*. However, in response to elevated ER stress, GRP78 is highly expressed in the ER disproportionately to KDEL-R1^17–19^, resulting in translocation of GRP78 to the cell surface in a highly cancer specific manner and in a broad range of solid tumors and hematological malignancies^18,19^. These characteristics make it an attractive therapeutic target^20,21^, that has only been evaluated in a limited manner for AML ^22^.

Here, we show the feasibility of targeting cell surface GRP78 in AML with CAR T cells. We determine the expression of GRP78 by gene expression analysis and flow cytometry, and have designed a panel of GRP78-specific CARs (GRP78-CARs) using a peptide that specifically binds to GRP78^23^. We demonstrate that GRP78 is overexpressed in a broad range of AML samples. Despite GRP78-CAR T cell cultures showing evidence of minimal fratricide and antigen-dependent T cell differentiation, they have potent anti-AML activity without HPC toxicity. Finally, preventing antigen-dependent T cell differentiation with dasatinib during GRP78-CAR T cell production further enhances their efficacy.

## Results

### GRP78 is expressed on the cell surface on primary AML blasts and PDX samples

**Fig. 1:**
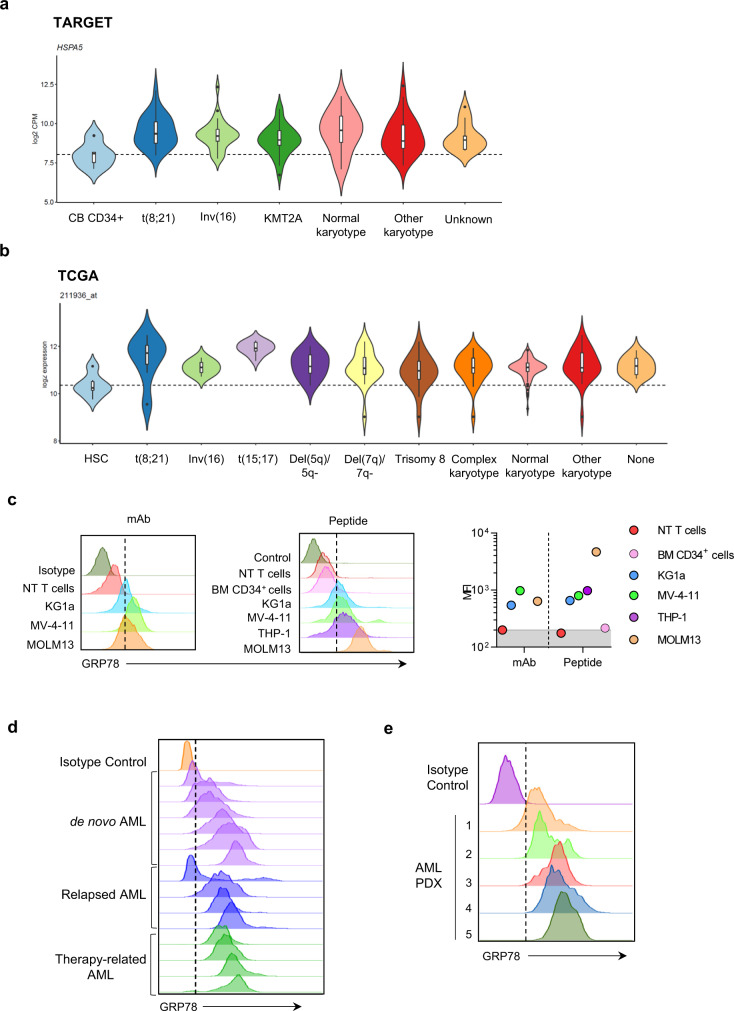
GRP78 is expressed on the cell surface on primary AML blasts and PDX samples. **a** Violin plots of gene expression analysis by RNA seq comparing HSPA5 expression on normal cord blood CD34+ cells to the TARGET dataset AML samples (*N* = 159). **b** Violin plots of microarray data comparing normal control HPCs to the TCGA (*N* = 244) dataset AML samples. Statistical analysis: TARGET and TCGA datasets, normal controls (CD34+ cells/HPCs) vs AML; *T*-test with pairwise comparisons was used, *p*-value < 0.05. The properties of the box-plots are defined as follows; minima: lower whisker = smallest observation greater than or equal to lower hinge − 1.5 * IQR (IQR = interquartile range: the difference between the 75th and 25th percentiles), box lower hinge = 25% quantile, box middle = median, 50% quantile, box upper hinge = 75% quantile, maxima: upper whisker = largest observation less than or equal to upper hinge + 1.5 * IQR. **c** Flow cytometric analysis showing cell surface GRP78 expression on normal NT T cells and normal bone marrow (BM) CD34+ cells^56^ and AML cell lines (left panel). Mean Fluorescence Intensity (MFI) of mAb (10C3 Dylight488 Ab) compared with Peptide (Biotin-Ahx-CTVALPGGYVRVC) staining in AML cell lines and healthy controls (right panel). **d** Flow cytometric analysis of 14 primary AML samples showing cell surface GRP78 expression (de novo *N* = 6, relapsed *N* = 4, therapy related *N* = 4). **e** Flow cytometric analysis of 5 AML-PDX samples showing percentage of cell surface GRP78+ cells. Source data are provided as a Source data file.

To demonstrate that GRP78 is overexpressed in AML blasts by gene expression analysis, we utilized samples from three publicly available databases (RNAseq from TARGET^24^(pediatric): *N* = 159; microarray data from TCGA^25^ (adult): *N* = 244 and MILE study^26^ (adult and pediatric): *N* = 252, downloaded from Bloodspot^27^ (Fig. 1a, b and Supplementary Fig. 1) in comparison to cord blood (CB) for RNAseq or HPC from adult bone marrow^28^ for microarray. GRP78 was differentially overexpressed in pediatric AML blasts, independently of the underlying mutation in all samples (TARGET: AML subgroup vs. CB CD34+ *p* < 0.05) (Fig. 1a). Likewise, GRP78 was expressed at significant higher levels in AML blasts of all subtypes present in the MILE study (*p* < 0.0001) (Supplementary Fig. 1a) and TCGA database (*p* < 0.05) excluding the Trisomy 8 TCGA subgroup (*p* = 0.0875) (Fig. 1b). To demonstrate cell surface expression of GRP78, we performed flow cytometry of AML cell lines (KG1a, MOLM13, THP-1, MV4-11) using an antibody specific for the ER retention sequence (KDEL) at the C-terminus of GRP78 or a biotin-conjugated peptide (Biotin-Ahx-CTVALPGGYVRVC) that specifically binds GRP78^23^. All AML cell lines expressed higher levels of cell surface GRP78 in comparison to non-transduced (NT) T cells and normal CD34+ adult bone marrow cells (Fig. 1c). Additionally, we examined cell surface GRP78 expression in B-cell malignancies Daudi (Burkitt’s lymphoma) and BV173 (Ph+ B-ALL) and found that there was GRP78 surface expression present on these cell lines, albeit to a lesser extent than on AML cell lines (Supplementary Fig.1b). Likewise, GRP78 was not expressed on the cell surface of T cells, granulocytes, monocytes, NK cells, NKT cells, and bone marrow progenitors (HSCs) (Supplementary Fig. 2a, b). We further demonstrated that GRP78 is highly expressed on the cell surface of >50% of 14 primary AML samples screened (de novo: *N* = 6, relapsed: *N* = 4, therapy-related: *N* = 4) (Fig. 1d, Supplementary Table. 1), and in all 5 AML PDX samples (Fig. 1e). We also evaluated the cell surface expression of GRP78 on leukemic stem cells (LSCs) in 13 primary AML samples (Supplementary Fig. 3a, b), demonstrating cell surface GRP78 expression on 2–51% of LSCs.

### Generation and characterization of GRP78-CAR T cells

**Fig. 2:**
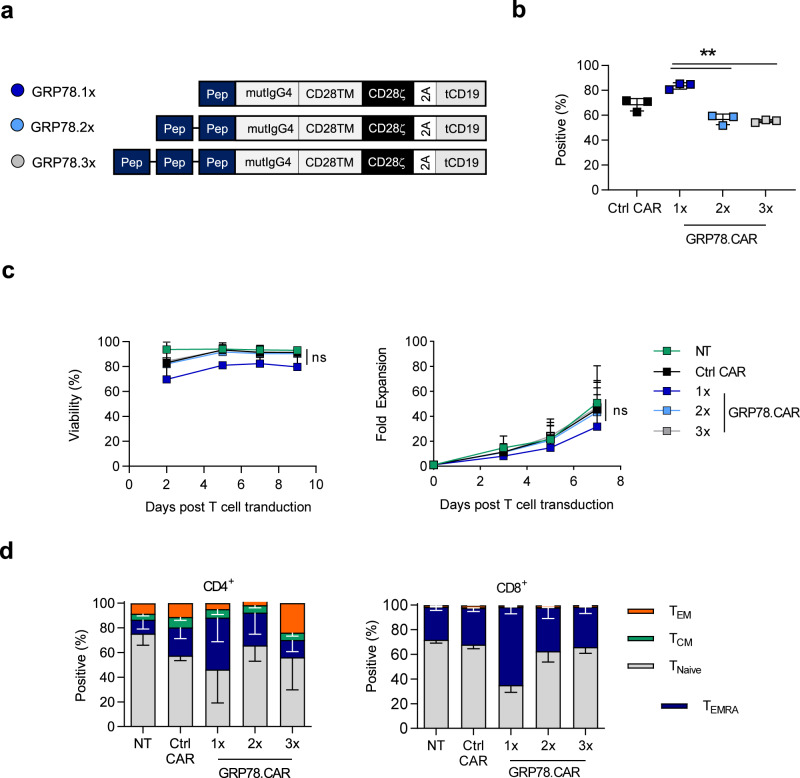
Generation and characterization of GRP78-CAR T cells. **a** Scheme of retroviral vectors encoding GRP78.1x-, GRP78.2x-, and GRP78.3x-CARs (‘Pep’ stands for peptide). **b** Transduction efficiency of GRP78-CAR constructs probing for tCD19 using flow cytometry on days 5–7 after retroviral transduction (control CAR: FRP5-CAR) (*N* = 3, 1x vs 2x *p* = 0.0008, 1x vs 3x *p* < 0.0001, 2x vs 3x *p* = 0.6131, two-sided *T*-test for pairwise comparisons was used). **c** Cell viability of CAR T cells measured from day 2 to day 9 post transduction using trypan blue (left panel, *N* = 3, *p* = ns, two-sided *T*-test for pairwise comparisons was used). Expansion of NT, Control CAR or GRP78 peptide CAR T cells measured for 7 days after transduction (right panel, *N* = 3, p = ns, two-sided *T*-test for pairwise comparisons was used). **d** Flow cytometric analysis of immunophenotype of GRP78 CAR transduced cells on day 7 post transduction (EM: CCR7−, CD45RA−; CM: CCR7+, CD45RA−; Naive-like: CCR7+CD45RA+; T_EMRA_: CCR7-, CD45RA+) (*N* = 3, *T*-test was used with pairwise comparisons, *p* < 0.05). The data in Fig. 2 are presented as mean values ± SD. Source data are provided as a Source data file.

To target cell surface GRP78-positive AML cells, we designed GRP78-CARs with one (1x), two (2x), or three (3x) copies of the GRP78-specific peptide^23^, which we used for flow cytometry analysis, as an antigen recognition domain (GRP78.1x-, GRP78.2x-, GRP78.3x-CAR). The CAR backbone was identical for all three CARs and consisted of a mutated IgG4 hinge^29^, a CD28 transmembrane domain, a CD28 costimulation domain, and a CD3ζ activation domain. These CARs were cloned into a retroviral vector upstream of a T2A sequence and truncated CD19 (tCD19) tag (Fig. 2a). GRP78.1x-CAR T cells had the highest median transduction efficiency as judged by tCD19 expression, followed by GRP78.2x-CAR, and GRP78.3x-CAR (Fig. 2b). Higher levels of GRP78.1x-CARs were also confirmed by Western blot analysis (Supplementary Fig. 4a). While GRP78.1x-CAR T cell expansion and viability were slightly lower than for the other CAR constructs, this did not reach statistical significance (Fig. 2c). We observed transient expression of low levels of cell surface GRP78 on T cells as judged by mean fluorescent intensity (MFI) after activation (Supplementary Fig. 4b, c), and the decrease in viability was blocked if GRP78-CAR T cells were generated in media that contained free GRP78-specific peptide (Supplementary Fig. 4d).

All GRP78-CAR T cell populations had a CD4:CD8 ratio of ~1:2 (Supplementary Fig. 4e), and CD4^+^ T cell subsets of all GRP78-CAR T cell populations and controls (NT, Control CAR) and CD8^+^ T cells of GRP78.2x- and GRP78.3x-CAR T cells displayed a predominantly naive-like (CCR7^+^ CD45 RA^+^) phenotype. However, GRP78.1x-CAR CD8^+^ T cells showed a predominantly differentiated effector memory (CCR7^-^ CD45RA^+^) T_EMRA_ phenotype (NT vs GRP78.1x-CAR T cells: *p* = 0.0005) (Fig. 2d). However, GRP78.1x-CAR CD8^+^ T cells did not display signs of T cell exhaustion as judged by TIM3, PD1, or LAG3 expression (Supplementary Fig. 5a, b).

### GRP78-CAR T cells recognize AML cells expressing cell surface GRP78 in vitro

**Fig. 3:**
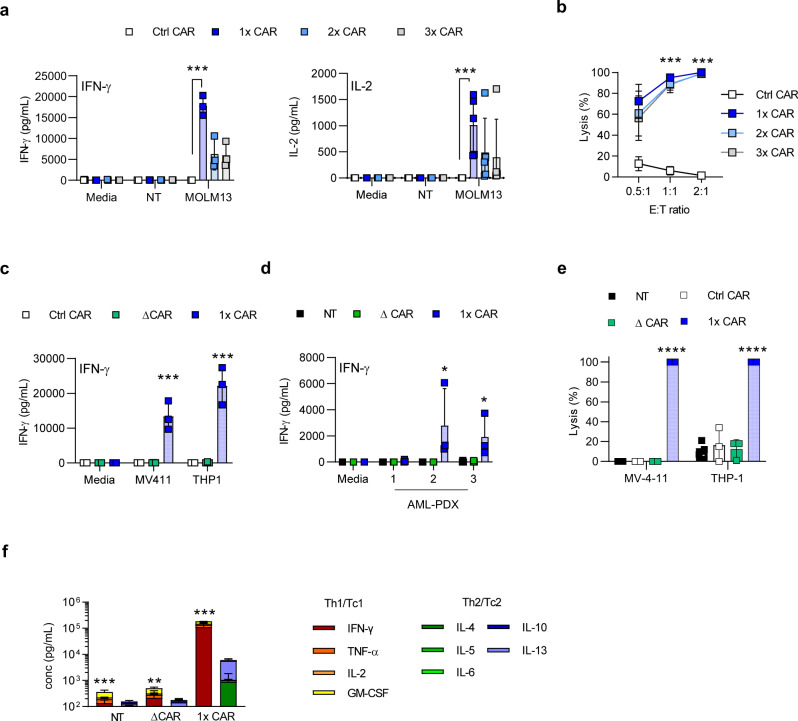
GRP78-CAR T cells target AML cells expressing cell surface GRP78 in vitro. **a** Determination of cytokine secretion by ELISA. Effector cells (GRP78-CARs or control CAR) were cocultured with media, NT T cells (GRP78− target) or MOLM13 (GRP78+ target) at a 2:1 E:T ratio. Supernatants were harvested 24 h later. We assessed IFNγ or IL-2 secretion using an ELISA assay (GRP78.1x CAR vs control CAR; IFN-γ: *N* = 3 *p* = 0.002, IL-2: *N* = 5 *p* = 0.0016, Generalized linear model was used to test group differences). **b** Luciferase-based cytotoxicity assay of GRP78-CAR T cells or control effector T cells (NT T cells, Control CAR or ΔCAR) against MOLM13 cells at 3 different E:T ratios (*N* = 3, for the ratio 2:1 *p* = 0.0003, for 1:1 *p* = 0.002, for 0.5:1 *p* = 0.01 Generalized linear model was used to test group differences). **c** Determination of IFN-γ secretion by ELISA in coculture assays with MV-4-11 (GRP78.1x-CAR vs Δ-CAR; IFN-γ *N* = 3, two-sided *T*-test was used with pairwise comparisons, *p* = 0.03, two-sided *T*-test was used with pairwise comparisons) or THP-1 (GRP78.1x-CAR vs Δ-CAR; IFN-γ *N* = 3, *p* = 0.01 two-sided *T*-test was used with pairwise comparisons). **d** Three AML PDX samples were cocultured with GRP78. 1x-CAR, Δ-CAR or NT T cells for 24 h at a 1:1 E:T ratio and IFN-γ secretion was determined (*N* = 3, GRP78.1x CAR vs Δ-CAR, two-factor ANOVA, *p = −0.01). **e** Luciferase-based cytotoxicity assay of GRP78-CAR T cells or control effector T cells (NT T cells, Control CAR or ΔCAR) or Control CAR T cells against MV-4-11 and THP-1 AML cell lines at a 2:1 E:T ratio. (GRP78.1x-CAR vs Δ-CAR; *N* = 3, *T*-test was used with pairwise comparisons, MV-4-11 *p* < 0.001, THP-1 *p* = 0.004). **f** Multiplex analysis of cytokine production by GRP78-CAR T cells or control effector T cells (NT T cells or ΔCAR) against MV-4-11 cells at a 1:1 E:T ratio. Supernatant for ELISA was collected 24 h. after stimulation (*N* = 3, *T*-test, NT *p* = 0.0006, ΔCAR *p* = 0.0024, 1x-CAR *p* = 0.00036). The data in Fig. 3 are presented as mean values ±  SD. Source data are provided as a Source data file.

To determine GRP78-CAR T cell effector function, we co-cultured T cells expressing the different GRP78-CARs with cell surface GRP78^+^ MOLM13 or cell surface GRP78^-^ target cells (NT T cells) at an effector:target (E:T) ratio of 2:1. HER2-CAR T cells served as negative controls (Ctrl CAR T cells). IFN-γ or IL-2 concentrations in culture media were measured by ELISA after 18–24 h. GRP78.1x-CAR T cells produced significantly more IFN-γ and IL-2 in comparison to Ctrl CAR T cells in the presence of MOLM13 (Fig. 3a). No cytokine production was observed in the presence of GRP78-negative target cells (NT T cells) or media. Antigen specificity was confirmed using a luciferase-based cytotoxicity assay (Fig. 3b). Previous reports have shown that targeting cell surface GRP78 with an antibody or a peptide can induce apoptosis of tumor cells^30,31^. To exclude that simple CAR binding to cell surface GRP78 contributes to tumor cell killing, we designed a non-functional GRP78.1x-CAR (GRP78.ΔCAR) that lacked the CD28 costimulation and CD3ζ activation domains but was otherwise identical to the GRP78.1x-CAR. GRP78.ΔCAR T cells co-cultured with cell surface GRP78+ MOLM13 cells (Supplementary Fig. 6a–c) did not exhibit any antitumor activity, confirming that cell surface GRP78 binding alone is insufficient to induce tumor cell death (Ctrl CAR vs GRP78.ΔCAR, *p* = NS). Next, we extended our in vitro studies to other GRP78^+^ AML cell lines (MV-4-11, THP-1). GRP78.1x-CAR T cells produced significantly increased amounts of IFN-γ in the presence of MV-4-11 and THP-1 AML cells in comparison to GRP78.ΔCAR and Ctrl CAR T cells (Fig. 3c). We further demonstrated that GRP78.1x-CAR T cells recognized AML PDX samples as judged by significant IFN-γ production in contrast to GRP78.ΔCAR and NT T cells (Effectors) (Fig. 3d). In addition, MV-4-11 and THP-1 AML cells were recognized and killed by GRP78.1x-CAR T cells in a luciferase-based cytotoxicity assay (Fig. 3e). To determine if GRP78.1x-CAR T cells secrete other cytokines than IFN-γ and IL-2, we co-cultured MV-4-11(Target cells) with NT, GRP78.ΔCAR, or GRP78.1x-CAR T cells (Effectors) and determined the expression of Th1/Tc1 (IFN-γ, TNF-α, GMCSF, IL-2) and Th2/Tc2 (IL-4, IL-5, IL-6, IL10, IL13) cytokines. GRP78.1x-CAR T cells secreted significantly more Th1/Tc1 and Th2/Tc2 cytokines (Fig. 3f). Thus, we have successfully generated GRP78-CAR T cells that produce cytokine and kill AML cells in an antigen-dependent manner.

### GRP78-CAR T cells sequentially kill tumor cells and secrete cytokines

**Fig. 4:**
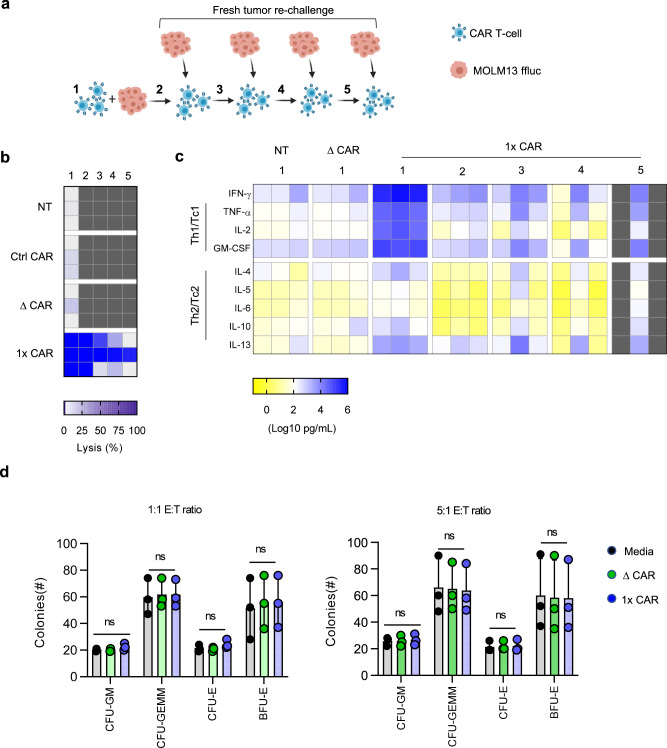
GRP78-CAR T cells sequentially kill tumor cells and secrete cytokines and do not target normal HPCs. **a** Schematic for serial stimulation assay using MOLM13 ffluc. cells. **b**, **c** Serial stimulation assay using effector T cells (GRP78.1x-CAR, control CAR, ΔCAR or NT T cells) and MOLM13 cells. Fresh MOLM13 cells were added every 72 h (*N* = 3). **b** Luciferase-based cytotoxicity assay (*N* = 3). **c** Multiplex analysis of cytokine production by GRP78-CAR T cells or control effector T cells (NT T cells or ΔCAR) against MOLM13 cells at a 1:1 E:T ratio. Supernatant for ELISA was collected 24–72 h. after stimulation (*N* = 3, *T*-test, *p* < 0.001). **d** CFU assay: Effector T cells were incubated with healthy adult HPCs for 4 h at E:T ratios of 1:1 and 5:1, plated on semisolid media, and BFU-E (burst forming unit—erythroid) and CFU colonies (colony-forming unit—erythroid: CFU-E, colony-forming unit—granulocyte, erythroid, macrophage, megakaryocyte: CFU-GEMM), were enumerated after 12–14 days (*N* = 3, technical replicates; one-way ANOVA and *T*-test for pairwise comparison; *p = 0.0109; ns; not significant). The data in (**d**) are presented as mean values ± SD. Source data are provided as a Source data file.

To evaluate if GRP78.1x-CAR T cells can sequentially kill AML cells and produce cytokines we performed a repeat stimulation assay in which NT, GRP78.ΔCAR, or GRP78.1x-CAR T cells (Effector cells) were stimulated every 3 days with MOLM13.GFP.ffluc cells (Target cells) (Fig. 4a). Before re-stimulation, we evaluated percentage of viable tumor cells by performing a luciferase assay and collected co-culture supernatant for cytokine analysis. Depending on the donor, GRP78.1x CAR T cells killed tumor cells between 2 and 5 times (Fig. 4b). GRP78.1x-CAR T cells also consistently produced Th1/Tc1 (IFN-γ, TNF-α, GMCSF, and/or IL-2) cytokines for at least 3 stimulations, although there was a significant decrease with each stimulation (Fig. 4c). While GRP78.1x-CAR T cells also produced Th2/Tc2 (IL-4, IL-5, IL-6, IL10, and/or IL13) cytokines, expression was significantly lower than Th1/Tc1 cytokines (Fig. 4c), confirming our previous findings with MV-4-11 cells.

### GRP78-CAR T cells do not target normal HPCs

To interrogate the effects of GRP78-CAR T cells on normal HPCs, we performed standard colony-forming unit (CFU) assays to measure the number of BFU-E, CFU-E, CFU-GM, and CFU-GEMM post exposure to GRP78.1x or GRP78.2x-CAR T cells at an E:T ratio of 1:1 or 5:1. Media and GRP78.ΔCAR T cells served as controls for both CFU assays; in addition NT T cells served as an effector control for the GRP78.2x-CAR T cell CFU assay. After 12 to 14 days CFUs were enumerated, and we observed no significant difference between GRP78.1x vs GRP78.ΔCAR or GRP78.2x vs GRP78.ΔCAR T cells (Fig. 4d and Supplementary Fig. 7). GRP78.2x-CAR T cells induced a decrease in CFU-Es in comparison to NT T cells at an E:T ratio of 5:1, but this was not confirmed for BFU-Es. Thus, neither GRP78.1x- or GRP78.2x-CAR T cells recognize normal HPCs

### GRP78-CAR T cells have potent anti-AML activity in vivo

**Fig. 5:**
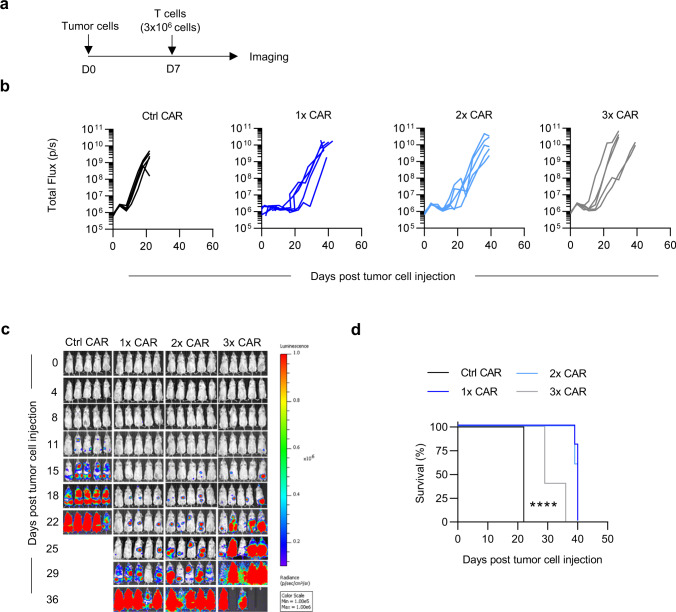
GRP78-CAR T cells have potent anti-AML activity in vivo. **a** Schematic of in vivo experimental design. **b**. MOLM13 xenograft model. NSG mice were injected with MOLM13 (5x10^3^) cells i.v. (tail vein), and on day 7 received a single i.v. dose of 3 × 10^6^ T cells. IVIS imaging was performed twice weekly. **c** Bioluminescence data (total flux = photons/s) **d** Survival curve (*N* = 5, *p* < 0.005, Log-rank test). Source data are provided as a Source data file.

**Fig. 6:**
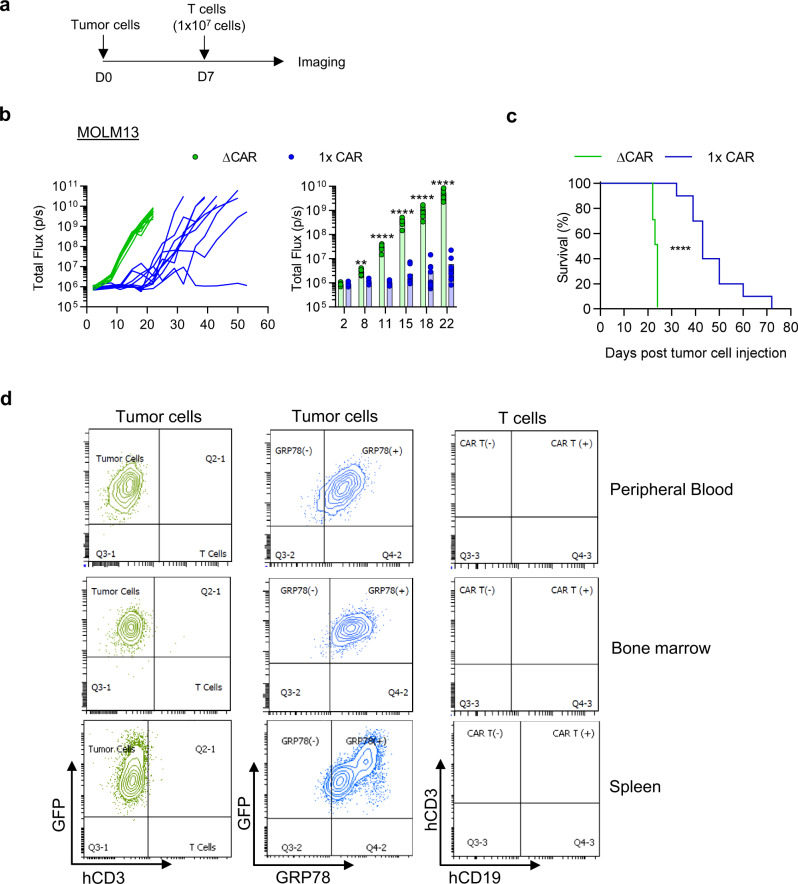
GRP78-CAR T cells have potent anti-AML activity in vivo. **a** Schematic of the in vivo experimental design. **b** NSG mice were injected with MOLM13 (5 × 10^3^) cells i.v. (tail vein), and on day 7 received a single i.v. dose of 1 × 107 T cells. IVIS imaging was performed twice weekly (*N* = 10, **p = 0.001, ****p < 0.0001, two-way ANOVA with multiple comparisons). The data in (**b**) are presented as mean values ± SD. **c** Survival curve (*N* = 10, *p* < 0.0001, Log-rank test). **d** Peripheral blood, spleen, and bone marrow was collected from mice on days 39–45 post tumor injection. Single cell suspensions were stained for GRP78, hCD3, and hCD19 and analyzed by flow cytometry to determine the presence of cell surface GRP78^+^ MOLM13 cells as well as CAR T cells; representative dot plots are shown. Source data are provided as a Source data file.

To test the efficacy of the GRP78-CAR T cells in vivo we first compared the antitumor activity of one IV dose of 3 × 10^6^ GRP78.1x-, GRP78.2x- or GRP78.3x-CAR T cells in the AML MOLM13.GFP.ffluc NSG xenograft model (Fig. 5a–d). NSG mice were injected with 5×10^3^ MOLM13.GFP.ffluc cells on day 0, followed by CAR T cells on day 7. While all GRP78-CAR T cell populations had significant anti-AML activity in comparison to control CAR T cells, GRP78.1x-, and GRP78.2x-CAR T cells were more efficacious than GRP78.3x-CAR T cells. We then escalated the cell dose to 1 × 10^7^ and compared GRP78.1x- vs GRP78.ΔCAR T cells (Fig. 6a–d). Only GRP78.1x-CAR T cells had significant antitumor activity, demonstrating that the in vivo anti-AML activity depends on the expression of functional GRP78-CARs on T cells. While GRP78-CAR T cell treated mice had a significant increase in overall survival (OS) in comparison to mice that had received control- or GRP78.ΔCAR T cells (*p* < 0.0001), AML eventually progressed (Fig. 6c, Supplementary Fig. 8). Progression was due to lack of CAR T cell persistence and not the development of antigen loss variants, since recurrent AML cells continued to express cell surface GRP78, and we could not detect CAR T cells in the peripheral blood, bone marrow, and/or spleen at the time of relapse (Fig. 6d, for gating strategy see Supplementary Fig. 9). To determine the persistence of GRP78-CAR T cells in vivo, MOLM13-bearing mice received on day 7 a single dose of GRP78.1x-CAR or GRP78.ΔCAR T cells genetically modified to express GFP.ffluc (Supplementary Fig 10a–c). GRP78.1x-CAR T cells showed significant early expansion compared to GRP78.ΔCAR T cells (*p* < 0.0001), followed by steady contraction through day 8 post injection. We observed no acute toxicity post GRP78-CAR T cells, which is reassuring since murine and human GRP78 are highly conserved and human GRP78-CAR T cells kill murine AML cell line C1498, which express GRP78 on their cell surface (Supplementary Fig. 11a–c).

### Dasatinib improves effector function of GRP78-CAR T cells in vitro and in vitro

**Fig. 7:**
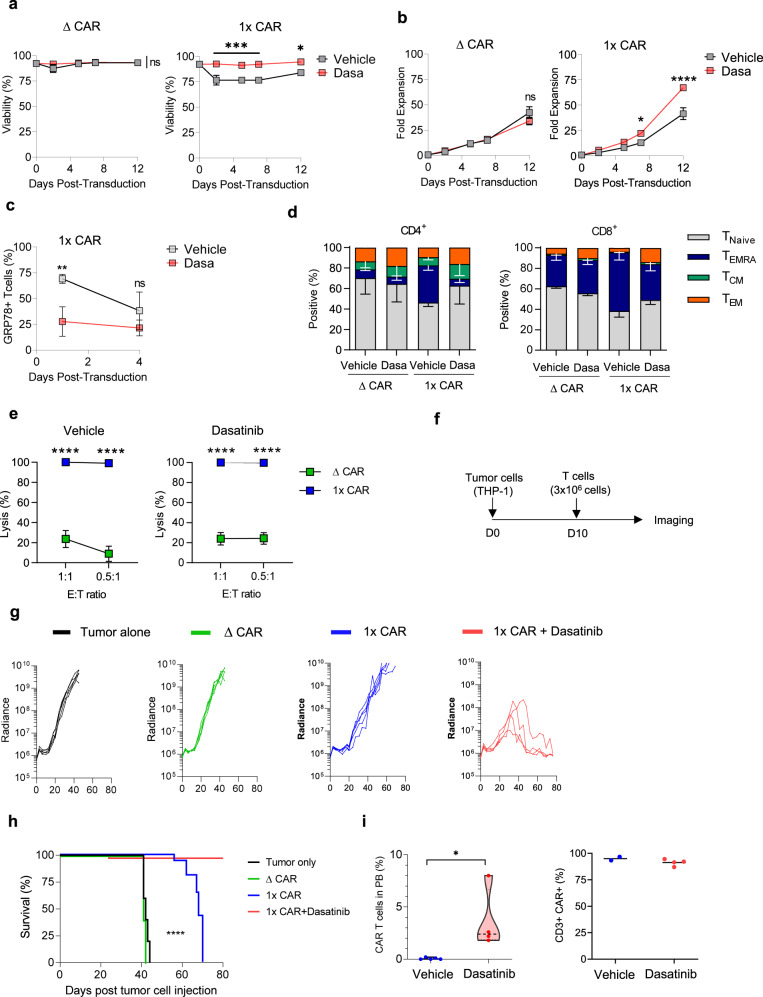
Dasatinib significantly improves the effector function of GRP78-CAR T cells. **a** Cell viability from day 0 to day 9 post retroviral transduction (*N* = 3, Left panel, GRP78.Δ-CAR vehicle vs dasatinib, *p* = ns; right panel, GRP78.1x-CAR, vehicle vs dasatinib, ***p < 0.001, *p = 0.05, Two-way ANOVA). **b** Expansion of GRP78.CAR-T cells for 12 days post transduction, (*N* = 3, Left panel, GRP78.Δ-CAR vehicle vs dasatinib, *p* = ns; right panel, GRP78.1x-CAR, vehicle vs dasatinib, ****p < 0.0001, *p < 0.05, Two-way ANOVA). **c** Cell surface GRP78 expression of GRP78.1x-CAR T cells on days 1 and 4 post transduction. (*N* = 3, vehicle vs dasatinib, Day 1, **p < 0.01; Day 4, *p* = ns, two-way ANOVA with multiple comparisons). **d** Flow cytometric analysis of GRP78-CAR T cell immunophenotype treated with vehicle or dasatinib on day 7 post transduction (EM: CCR7−, CD45RA-; CM: CCR7+, CD45RA−; Naive-like: CCR7+ CD45RA+; T_EMRA_: CCR7−, CD45RA+) (*N* = 3, GRP78.1x-CAR T_EMRA_ subset, vehicle vs dasatinib, paired T-test CD4+, *p* < 0.01, CD8+, *p* < 0.01). **e** Luciferase-based cytotoxicity assay of GRP78-CAR T cells or ΔCAR treated with vehicle (left panel) or dasatinib (right panel) against THP-1 cells at 2 different E:T ratios (*N* = 3, for ratios 1:1 and 0.5:1, *p* < 0.0001, two-way ANOVA with multiple comparisons). **f** Schematic of the in vivo experimental design. **g** NSG were injected on day 0 with 3 × 10^6^ THP-1.ffluc cells i.v. (tail vein) and on day 10 a single i.v. dose of 3 × 10^6^ T cells was injected. IVIS imaging was performed twice weekly (*N* = 5, *T*-test, for 1× CAR *p* = 0.01, for 1× CAR + Dasatinib *p* < 0.0001). **h** Survival curve (*N* = 5, *p* < 0.0001, Log-rank test). **i** Left panel: Percent CAR T cells in peripheral blood (PB) evaluated by flow cytometry on day 54 post tumor cell injection (day 44 after T cell infusion). (GRP78.1x-CAR, vehicle *N* = 5, dasatinib *N* = 4, two-tailed *T*-test, *p* = 0.02). Right panel: graph showing CD3+ CAR+ cells. The data in (**c**–**e**) are presented as mean values ± SD. Source data are provided as a Source data file.

To improve the effector function of GRP78-CAR T cells we first replaced the CD28 costimulatory domain in our CAR with 41BB (41BB.GRP78-CAR). While 41BB.GRP78-CARs were functional, they did not improve the ability of GRP78-CAR T cells to kill tumor cells in our restimulation assay (Supplementary Fig. 12a–e). Based on these findings, we did not evaluate 41BB.GRP78-CAR T cells in vivo. Since we had observed minimal fratricide and antigen-dependent T cell differentiation during GRP78-CAR T cell generation, we examined the levels of GRP78 on the surface of T cells post activation. We next investigated if blocking cell surface GRP78 expression and CAR signaling during CAR T cell production would improve the effector function of GRP78.1x-CAR T cells. Dasatinib treated GRP78.1x-CAR T cells had increased viability resulting in improved expansion and expressed lower levels of cell surface GRP78 post transduction in comparison to their vehicle (DMSO) treated counter parts (vehicle vs dasatinib: viability: *p* < 0.001; expansion: *p* < 0.0001; GRP78 cell surface expression: *p* < 0.01; Fig. 7a–c). Immunophenotypic analysis of dasatinib treated GRP78.1x-CAR T cells showed an increase in naive-like (CCR7^+^/CD45RA^+^) and a decrease in differentiated effector memory (CCR7^−^/CD45RA^+^) subsets when compared to vehicle treated GRP78.1x-CAR T cells (*p* < 0.01; Fig. 7d). In coculture assays, dasatinib treated GRP78.1x-CAR T cells had robust antitumor activity against THP-1 and MOLM13 cells (Fig. 7e, Supplementary Fig. 13a). In our repeat stimulation assay against MOLM13 cells, dasatinib treated GRP78.1x CAR T cells were able to maintain antitumor activity for up to 12 stimulations, while standard GRP78.1x CAR T cells only had antitumor activity for up to 6 stimulations. (Supplementary Fig. 13b).

To test the efficacy of dasatinib treated GRP78.1x-CAR T cells in vivo, we used the THP-1 AML model. NSG mice were injected with 3 × 10^6^ THP-1.GFP.ffluc cells on day 0, followed by 3 × 10^6^ GRP78-CAR T cells on day 10. Mice treated with GRP78.ΔCAR T cells showed no antitumor activity when compared to untreated mice (*p* = ns; Fig. 7g). Vehicle treated GRP78.1x-CAR T cells had transient antitumor activity and extended OS by up to 30 days (GRP78.ΔCAR vs. GRP78.1x-CAR: *p* < 0.0001; Fig. 7g, h). In contrast, dasatinib treated GRP78.1x-CAR T cells induced complete remission resulting in a significantly improved OS compared to mice that had received vehicle treated GRP78-CAR T cells (GRP78.ΔCAR vs. GRP78.1x-CAR: *p* < 0.0001; vehicle treated vs. dasatinib treated GRP78.1x-CAR, *p* < 0.05; Fig. 7g, h). This improved antitumor activity correlated with increased CAR T cell persistence of dasatinib treated GRP78.1x-CAR T cells on day 54 post tumor injection (day 44 post T cell infusion) (Fig. 7I).

## Discussion

Here we report on the expression of GRP78 in AML blasts, and the design and characterization of CAR T cells targeting GPR78. Collectively, our data demonstrate that GRP78 is expressed on the cell surface of AML blasts, and that GRP78-CAR T cells recognize and kill cell surface GRP78-positive AML blasts while sparing normal HPCs, myeloid cells and lymphocytes. In addition, GRP78-CAR T cells had potent antitumor activity in vivo.

Finding CAR T cell therapy targets for AML has been challenging due to the heterogeneity of AML and the overlapping expression of antigens on healthy tissues such as hematopoietic progenitor cells (HPCs) and AML blasts^32,33^. Preclinical and early clinical studies using CAR T cells for AML have focused on antigens such as CD33, CLL1, and CD123^32^. These antigens, however, are also expressed either on normal HPCs or mature neutrophils. GRP78, the master UPR regulator and ER chaperone is overexpressed and translocated to the cell surface in response to elevated ER stress in a broad range of solid tumors and hematological malignancies but not in normal tissues^20,21,34^. While there is a possibility that GRP78 is upregulated in noncancerous cells under certain disease states, it is important to note that elevation of cell surface GRP78 in any cell is a direct result of elevated ER stress. In the case of normal tissues, this elevation of ER stress also signals apoptosis which leads to rapid elimination of these cells^35,36^. This makes GRP78 an attractive target for CAR T cell therapy since CARs only recognize cell surface antigens in contrast to conventional αβ T cell receptors^1^.

GRP78 has been recognized for its role in leukemogenesis, providing pro-survival signals and serving as a growth co-receptor^37^. Differential cell surface expression of GRP78 between normal cells and AML blasts has been previously described albeit for a limited number of samples^22,37,38^. Here we extend these studies by demonstrating that GRP78 is also overexpressed in a broad range of adult and pediatric AML samples as judged by analyzing the TARGET, TCGA, and MILE datasets. However, overexpression alone does not predict cell surface expression of GRP78, and we, therefore, performed flow cytometry to demonstrate that GRP78 is consistently expressed on the cell surface of AML cell lines and primary AML blasts. Clearly, a larger number of AML samples need to be evaluated for GRP78 cells surface expression on LSCs in the future before definitive conclusions can be drawn. However, limited expression of GRP78 should not prevent further development of GRP78-CAR T cell therapy for AML. For example, of three targets (CD123, CD33, CLL-1) that are actively being explored in early phase clinical studies, only CD123 and CD33 are consistently expressed on LSCs at relapse (CD33: 95.3%; CD123: 92.7%) where as CLL-1 is not (20%)^39^. Despite variable expression of CLL-1 on LSCs, multiple early phase clinical studies targeting CLL-1 with CAR T cells are in progress (NCT04789408, NCT04884984, NCT04219163, NCT04923919). Of interest, interim results, of one CLL-1-CAR T cell therapy clinical study demonstrated that 3/3 patients with chemorefractory/chemoresistant AML achieved minimal residual disease negative disease complete responses after infusion of CLL-1-CAR T cells^40^. Likewise, several studies have highlighted that to achieve an effective AML-specific CAR T cell therapy, multiple antigens have to be targeted to accomplish AML specificity, and long lasting remissions by bypassing immune escape^39,41^. Thus, if variable expression of GRP78 is confirmed on LSCs, GRP78-CAR T cell therapy could be combined with targeting an antigen that is expressed consistently on LSCs.

Most antigen recognition domains of CARs consist of single chain variable fragments (scFVs) derived from mAbs^1^. Here, we used a peptide-based antigen recognition domain. Peptides as antigen recognition domains have been successfully used by others^42,43^, and may have advantages including limited immunogenicity in comparison to scFVs since they are smaller in size. The peptide we used in our study, had been previously identified using an unbiased phage display library screen and its specificity has been extensively characterized^23,44,45^. To confirm the specificity of GRP78-CAR T cells we used target cells that did not express GRP78 as judged by using an antibody that is specific for the KDEL sequence at the C-terminus of GRP78. We also attempted to generate leukemia and solid tumor lines in which GRP78 is knocked out by CRISPR/Cas9-based gene editing in collaboration with St. Jude’s Center for Advanced Genome Engineering (CAGE). However, despite numerous attempts, we were unsuccessful, which is most likely explained by the fact that GRP78 (HSPA5) is considered a common essential gene according to the Cancer Dependency Map (https://depmap.org/portal/). We generated GRP78-CARs that had one, two, or three GRP78 binding domains. T cells expressing the GRP78.1x-CAR outperformed GRP78.2x- and GRP78.3x-CAR T cells, which is most likely explained by higher levels of CAR expression. However, other contributing factors such as steric hindrance of multiple antigen binding domains or differences in signaling of GRP78.1x-, GRP78.2x-, and GRP78.3x-CARs cannot be excluded.

GRP78-CAR T cells killed cell surface GRP78^+^ AML cells in vitro and produced predominant Th1/Tc2 cytokines. In addition, GRP78-CAR T cells also recognized 2 out 3 primary cell surface GRP78^+^ AML-PDX samples. The limited ability of one PDX sample (AML-PDX 1) to activate GRP78-CAR T cells is most likely explained by its poor viability post thaw. In repeat simulation assays, GRP78-CAR T cells had significant antitumor activity. However, their effector function declined with repeat stimulations, which is expected based on the experience with other CAR T cell populations in repeat stimulation assays^46,47^. Investigators have explored approaches to overcome this limitation including using FDA approved drugs and transgenic expression of cytokines, cytokine receptors or other signaling molecules^46–51^.

In vivo GRP78-CAR T cells had potent anti-AML activity resulting a significant survival advantage of treated mice; however, mice invariably presented with GRP78^+^ AML relapse. The absence of antigen loss variants is consistent with GRP78 biology since its translocation to the cell surface reflects elevated ER stress, which is driven by cellular processes that are critical for the malignant phenotype of tumor cells^52,53^. Thus, decreased cell surface expression of GRP78 in response to CAR T cell therapy is unlikely. Limited GRP78-CAR T cell persistence, which was responsible for tumor recurrences, may be attributed to the CD28 costimulatory domain of our GRP78-CAR^54^.

We used dasatinib to overcome the observed fratricide and early T cell activation of GRP78-CAR T cells. This resulted in improved effector function of GRP78-CAR T cells in vitro and in vivo. Our findings are consistent with previous studies that have shown that exposing T cells to dasatinib during CAR T cell production potentiate their persistence and functionality by blocking CAR signaling and T cell differentiation^55,56^. In addition, we demonstrate here that dasatinib reduces cell surface GRP78 expression post activation/transduction in T cells, which is most likely mediated through inhibition of Src family kinases by dasatinib^19,57,58^.

None of the mice that received functional GRP78-CAR T cells exhibited any overt toxicities, potentially indicating a favorable safety profile since murine and human GRP78 are highly conserved^21^. However, further work in immune competent animal models is needed to confirm this. Likewise, we demonstrated that GRP78-CAR T cells did not recognize HPCs in CFU assays, and early clinical testing of the GRP78-specific mAb, PAT-SM6, also demonstrated an encouraging safety profile in humans^59^.

In summary, we demonstrate here that GRP78 is expressed on the cell surface of AML cells, and generate for the first time a peptide-based GRP78-CAR. GRP78-CAR T cells have potent anti-AML activity in vitro and in vivo without overt toxicity. Thus, GRP78-CAR T cells warrant further exploration as an immunotherapeutic for patients with AML.

## Methods

The research in this study complies with all the relevant ethical regulations. All methods involving human subjects were carried out in accordance with the Declaration of Helsinki. Human peripheral blood mononuclear cells (PBMCs) from healthy donors were obtained under St. Jude Children’s Research Hospital (St. Jude) IRB approved protocols (PACT/Pro00008053; NR17-114), after acquiring informed consent. All in vivo studies were carried out following protocols approved by the Institutional Animal Care and Use Committee in accordance with the American Association for Laboratory Animal Science at St. Jude Children’s Research Hospital.

### Cell lines and culture methods

The following cell lines were procured from American Type Culture Collection (ATCC, Manassas, VA): 293T, KG1a, MV-4-11, THP-1 cell lines and Daudi. BV173 and MOLM13 cell lines were purchased from Leibniz Institute (DSMZ, German Collection of Microorganisms and Cell Cultures, Braunschweig, Germany). Murine cell line C1498 (ATCC, Manassas, VA). Bone marrow CD34+ HPCs were obtained from Lonza (Lonza Biosciences, Cat. No. 2M-101D). Fourteen primary pediatric AML samples (initial diagnosis, therapy-related or relapsed) were obtained from the St. Jude Children’s Research Hospital Biorepository as part of an IRB approved protocol. Five patient-derived xenograft (PDX) cell lines were established by intravenous (i.v.) injection of pediatric primary AML samples into NSG-S mice and subsequently propagated in vivo^60^. Additional details on the primary pediatric AML and PDX samples is provided in Supplementary Fig. 1. The generation of MOLM13 expressing an enhanced green fluorescence protein/firefly luciferase fusion protein (GFP.ffluc) was previously reported (MOLM13.GFP.ffluc)^61^. Cell lines were cultured in RPMI 1640 (ThermoFisher Scientific) or DMEM (GE Life Sciences) and grown in humidified incubators at 37 °C and 5% COR_2_R. All culture media was supplemented with 10% Fetal Bovine Serum (Thermo Scientific) and GlutaMAX (2 mmol/L; Invitrogen, Carlsbad, CA). Cell lines were authenticated using the ATCC’s human STR profiling cell authentication service and routinely checked for Mycoplasma by the MycoAlert Mycoplasma Detection Kit (Lonza).

### RNA-Seq read mapping, gene expression summary, and batch correction

We used our StrongARM pipeline to map RNA reads, as previously described^62^. BWA was used to align the Paired-end reads from RNA-seq to the following database files: (i) the human GRCh37-lite reference sequence, (ii) RefSeq, (iii) a sequence file representing all possible combinations of non-sequential pairs in RefSeq exons and, (iv) the AceView database flat file downloaded from UCSC representing transcripts constructed from human ESTs. STAR was used to map the paired-end reads to the human GRCh37-lite reference sequence. We aligned the mapping results from databases (ii)–(iv) to the human reference genome coordinates. The best of five alignments were selected for the construction of the final BAM file.

Reads from aligned bam files were assigned to genes and counted using HTSeq^63^ with the GENCODE human release 19 gene annotation and Log2 CPM (counts per million) values were generated. A cut-off of 10 counts was used to calculate the corresponding CPM, which was used as the threshold for expression. We first determined the number of samples in the smallest group among all groups being compared as *N* (*N* = 5 in this study). For a gene to be considered as expressed, we required that at least *N* = 5 samples to have CPM values greater than the above-mentioned expression threshold. Genes not meeting this cutoff were excluded from downstream analysis. The detected batch effect due to data source of St. Jude vs. TARGET was corrected using the ComBat method available from the R package SVA^64^. Limma R package^65^ was used for differential gene expression analysis.

### Generation of retroviral vectors

We synthesized cDNAs (GeneArt, ThermoFisher Scientific, Waltham, MA) encoding the IgG heavy chain leader sequence and one (1x), two (2x), of three (3x) copies of the GRP78-specific peptide CTVALPGGYVRVC)^23^. These were subcloned into a pSFG retroviral vector that encoded a mutant IgG4 hinge, a CD28 transmembrane domain, a CD28.CD3ζ signaling domain, a T2A ribosomal skip sequence and truncated CD19 (tCD19) to enable detection of transduced cells. The ΔGRP78.CAR was generated by deleting the CD28.CD3ζ signaling domain from the GRP78.1x-CAR. The generation of control-CARs (HER2-CAR.CD28.CD3ζ) have been previously reported^66,67^. The sequence of all cloned constructs was confirmed by sequencing performed by Hartwell Center DNA Sequencing Core at St. Jude Children’s Research Hospital with Big Dye® Terminator (v3.1) Chemistry on Applied Biosystems 3730XL DNA Analyzers (Thermo Fisher Scientific, Waltham). RD114-pseudotyped retroviral particles were generated as previously described ^61^.

### Generation of CAR T cells

All methods involving human subjects were carried out in accordance to the Declaration of Helsinki. Human peripheral blood mononuclear cells (PBMCs) from healthy donors were obtained under a St. Jude Children’s Research Hospital (St. Jude) IRB approved protocol, after acquiring informed consent. PBMCs were stimulated on CD3 (1 µg/mL, Miltenyi Biotec, Bergisch Gladbach, Germany) and CD28 (1 µg/mL, Miltenyi Biotec, Germany) antibody-coated, non-tissue culture treated 24-well plates (Corning, Corning, NY). Human interleukin (IL) 7 (10 ng/mL, Peprotech, Rocky Hill, NJ) and IL-15 (5 ng/mL, Peprotech) were added to cultures on day 2. On day 3, T cells were transduced with retroviral particles on RetroNectin (Takara Bio USA, Mountainview CA) coated plates in the presence IL-7 (10 ng/mL) and IL-15 (5 ng/mL). T cells were subsequently expanded with IL-7 and IL-15. Non-transduced (NT) T cells were activated with CD3/CD28 antibodies and expanded in parallel with IL7 and IL-15. Following expansion for 5–7 days the transduced cells were analyzed for CAR expression using flow cytometry and subsequently used for functional assays. To manufacture CAR T cells in the presence of dasatinib, dasatinib (30 nM; LC Labs. Catalog# D-3307. Woburn MA USA) was added to the culture medium at the time of transduction and was replenished every 48–72 h. DMSO was used as vehicle control. Dasatinib was removed at the time of in vitro and in vivo functional studies.

### Flow cytometric analysis

All antibodies were used at 1:50 dilution unless stated otherwise. Cell surface GRP78 was detected either by a KDEL antibody (Abcam, Clone-10C3; Cat. No. ab115638) or a GRP78-specific peptide with an N-terminal Biotin tag (Biotin-Ahx-CTVALPGGYVRVC) was custom synthesized by Genscript (Piscataway, NJ) and used at 3 μM working conc. in combination with Streptavidin PE at 1:100 dilution (BioLegend, San Diego, CA. Cat. No. 405204). The following antibodies were purchased from BD Biosciences: CD8 APC-H7 (Clone: SK1, BD Biosciences, San Jose, CA. Cat No.560179), CCR7 FITC (Clone: 150503, BD Biosciences, Cat No.561271), CD4-KrO (Clone:13B8.2 Beckman Coulter, Cat. No. A96417) CD45RA APC (Clone: HI100 BioLegend, Cat.No.304150), CD19 APC (Clone: J3-119 Beckman Coulter, Cat. No. IM2470U), CD19 PE (Clone: J3-119 Beckman Coulter, Cat. No. IM1285U), TIM3 PE-Cy7 (Clone: F38-2E2 BioLegend, Cat.No.345014), LAG3 PE 1:50 dilution (Clone:R&D Biosystems, Cat.No. FAB2319P), PD1 BV605 (Clone: EH12.2H7 BioLegend, Cat.No.329924). CD19-APC or CD19-PE antibodies were used to stain for tCD19 on GRP78-CAR constructs. Recombinant Human ErbB2/Her2 Fc Chimera Protein (R&D Systems, Cat. No 1129-ER) or Alexa Fluor® 647 AffiniPure Goat Anti-Mouse IgG, F(ab’)2 (Jackson Immunoresearch Laboratories, Cat. No. 115-605-006) 1:150 dilution were used to detect the HER2-CAR. 5×10^5^ transduced and NT T cells were washed with 1X PBS and the cells were incubated with 3 μL of antibody on ice and protected from light for 30 mins. LIVE/DEAD™ Fixable Aqua Dead Cell Stain for 405 nm excitation, 1:200 dilution (ThermoFisher Scientific eBioscience™ Cat. No. L34957) was used as a dead stain. Cells were washed with 1× PBS + 2% FBS. All samples were acquired on FACS Canto II or Lyric instruments (BD Biosciences). The analysis was performed using FlowJo 10.7.1 software (BD Biosciences). CD3 BUV395 (Clone: SK7 BD Biosciences, Cat.No. 564000), CD56 FITC (Clone: 5.1H11 Biolegend Cat.No. 362546), CD19 BV711(Clone: SJ25C1 BD Biosciences, Cat.No. 563036), CD14 PE-CY7 (Clone: 61D3 ThermoFisher Scientific, Cat.No. 25-0149-42), CD15 BUV737 (Clone: W6D3 BD Biosciences, Cat.No.741876), CD16 APC-H7 (Clone: 3G8 BD Biosciences, Cat.No. 560715), CD11b BUV563 (Clone: ICRF44 BD Biosciences, Cat.No.741357), CD33 BV786 (Clone: WM53 Biolegend Cat.No. 303427)were used to stain the cell lineages from peripheral blood. CD38 BV510 (Clone: HIT2 Biolegend Cat.No. 303530), CD123 BV421(Clone: 9F5 BD Biosciences, Cat.No.565928), CD45 FITC (Clone: 2D1 BD Biosciences, Cat.No. 347463), CD33 BV786 Biolegend Cat. No. 303427), CD34 APC (Clone: 563 BD Biosciences Cat.No. 561209), Live/Dead Fixable Viability Dye eFluor™ 455UV (ThermoFisher Scientific eBioscience™ Cat. No. 65-0868-18) were used to stain for LSCs on primary AML samples. The peripheral blood lineage and primary AML LSC panels were acquired using FACS Symphony and the analysis was performed using FACS Diva (BD Biosciences).

### Cytotoxicity assays

To determine the cytotoxic potential of the CAR T cells we utilized a luciferase-based cytotoxicity assay, NT or CAR T cells were co-cultured with 5x10^5^ GFP.ffluc expressing target cells at a 1:1 Effector: Target (E:T) ratio in a 96-well tissue culture plates overnight. In the luciferase-based assay, 100μl of MOLM13.GFPffluc cells were incubated with D-Luciferin. Luminescence was measured on a Tecan Infinite ® 200 (Life Sciences-Tecan, Männedorf, Switzerland) and analyzed using Magellan Software (Life Sciences-Tecan).

### Western blot

Cells were lysed using RIPA buffer and protease inhibitor cocktail (Roche complete Mini tablets). The samples were boiled for 7 min in 4x Laemmli Sample buffer (Cat. No. 1610747, Bio-Rad Laboratories). SDS Page was performed using Mini-PROTEAN® TGX™ Precast Gels and a Mini-PROTEAN Tetra Cell system (Bio-Rad laboratories). The proteins were transferred to a PVDF membrane (Millipore) and probed with primary antibodies at 1:1000 dilution (CD3z Clone-6B10.2: Cat. No. sc-1239; GAPDH Clone 6C5: Cat. No. sc-32233, Santa Cruz Biotechnology) and HRP-linked secondary antibody 1:2000 dilution (Amersham ECL Anti-mouse IgG, peroxidase-linked whole antibody, Cat. No. NA931V, GE Healthcare). The blots were developed using Clarity Western ECL Blotting Substrate (Cat. No. 1705060, Bio-Rad Laboratories) and imaged on the Odyssey® Fc Imaging System from LI-COR Biosciences and LI-COR Image Studio™ software version 5.2. Full uncropped blots shown in Source data file for Supplementary Figures.

### Cytokine ELISA

GRP78-positive (AML cell lines, PDX samples) or GRP78-negative (NT T cells) target cells were cocultured with effector cells at a 2:1 or 1:1 E:T ratio. As effector cells we used GRP78-CAR T cells or T cells that expressed a nonfunctional CAR (ΔGRP78.CAR) or CAR that recognized an irrelevant antigen (Ctrl CAR), or NT T cells. Following 24 h of co-culture, the supernatants were collected and IFN-γ and IL-2 levels were determined using ELISAs (R&D Systems) as per the manufacturer’s protocols.

### Repeat stimulation and cytokine multiplex assay

5x10^5^ effector T cells were plated at a 1:1 effector to target (E:T) ratio with GRP78+ target cells expressing firefly luciferase (MOLM13.ffluc) or at 2:1 effector to target (E:T) ratio with GRP78+THP-1. ffluc cells. Three days later, antitumor activity was determined by a luciferase-based assay and culture supernatants were collected. Afterward, E:T ratio was adjusted back to 1:1 or 2:1 by adding fresh tumor cells. Harvested culture supernatants were then analyzed using a custom human Cytokine/Chemokine Multiplex assay containing analytes for GM-CSF, IFN-γ, IL-10, IL-13, IL-2, IL-4, IL-5, IL-6, and TNF-α (EMD Millipore, Chicago, IL) as per manufacturer’s instructions.

### Colony-forming unit (CFU) assay

Media, GRP78.Δ, GRP78.1x-CAR, or GRP78.2x-CAR T cells were co-cultured with CD34^+^ bone marrow cells (Lonza, Basel, Switzerland) at an E:T ratio of 1:1 and 5:1 for 4 h and were then plated in the presence of MethoCult (Stemcell Technology, Vancouver, CA) in a 6-well SmartDish® (Stemcell Technology, Vancouver, CA), and incubated for 12-14 days at 37 °C. For the CFU assay with GRP78.2x-CAR T cells, NT T cells were also included as a control effector cell population. Plates were imaged using a Nikon C2 point-scanning confocal Microscope (Nikon, Tokyo, Japan) using a ×4 objective. BFU-E (burst Forming Unit—erythroid) and CFU colonies (colony-forming unit—erythroid: CFU-E, colony-forming unit—granulocyte, erythroid, macrophage, megakaryocyte: CFU-GEMM), and colony-forming unit—granulocyte, macrophage + CFU-GM) were enumerated.

### In vivo studies

In vivo studies were performed using NSG (NOD.Cg-Prkdcscid/Il2rgtm1Wjl/SzJ) mice obtained from St. Jude’s in-house breeding colony. The animals were on an automated 12-h on, 12-h off light cycle and the temperature and humidity was controlled and maintained at the room level with the help of an automated thermostat and humidistat (room temperature is set at 71 °F and relative humidity at 45%). 5 × 10^3^ MOLM13.GFP.ffluc or 3 × 10^6^ THP-1.GFP.ffluc cells were injected iv by tail vein injection. Tumor growth was monitored by twice weekly bioluminescence imaging using an IVIS®-200 imaging system (IVIS, Xenogen Corp., Alameda, CA) as previously described^66^. In vivo imaging was analyzed using Perkin Elmer’s Living Image^®^ software (version 4.5.5). Mice were euthanized at predefined endpoints or when they met euthanasia criteria in accordance with St. Jude’s Animal Resource Center.

### Statistical analysis

Descriptive statistics were calculated for all outcomes. The one or two factor ANOVA test was used to examine overall differences in outcomes between multiple constructs. (Comparisons across multiple groups were performed by one- or two-factor ANOVA when appropriate.) The overall test was followed by pairwise comparisons using *t*-test when appropriate (i.e., overall test *P* < .05). Generalized linear model was used to access the overall difference in outcomes with repeated measurements to account for intra subject correlation in each subject/donor. Log-rank test was used to test difference between constructs of all survival outcomes.

Statistical analyses were conducted with SAS 9.4. A two sided-significance level of *P* < 0.05 was used for all statistical test and adjustment for multiple testing was not performed due to small sample size and the exploratory nature of the analysis.

## Supplementary information

Supplementary Information

## Data Availability

The results published here are in part based upon data generated by the Therapeutically Applicable Research to Generate Effective Treatments (https://ocg.cancer.gov/programs/target) initiative, phs000218 (dbGaP substudy phs000465) and TCGA Research Network. TARGET AML data were obtained from the GDC Data Portal under accession phs000218. The data used for this analysis are available at https://portal.gdc.cancer.gov/projects and https://www.cancer.gov/tcga. The remaining data are available within the Article, Supplementary Information or Source data file. Source data are provided with this paper.

## Source data

Source data

## References

[1] June CH, Sadelain M (2018). Chimeric antigen receptor therapy. N. Engl. J. Med,.

[2] Shah NN (2020). CD4/CD8 T-cell selection affects chimeric antigen receptor (CAR) T-cell potency and toxicity: updated results from a phase I anti-CD22 CAR T-cell trial. J. Clin. Oncol..

[3] Raje N (2019). Anti-BCMA CAR T-cell therapy bb2121 in relapsed or refractory multiple myeloma. N. Engl. J. Med.

[4] Ramos, C. A. et al. Anti-CD30 CAR-T cell therapy in relapsed and refractory hodgkin lymphoma. *J. Clin. Oncol.* **8**, 3794–3804 (2020). 10.1200/JCO.20.01342 PMC7655020 32701411

[5] Maude SL (2018). Tisagenlecleucel in children and young adults with B-cell lymphoblastic leukemia. N. Engl. J. Med..

[6] Park JH (2018). Long-term follow-up of CD19 CAR therapy in acute lymphoblastic leukemia. N. Engl. J. Med..

[7] Turtle CJ (2016). CD19 CAR-T cells of defined CD4+:CD8+ composition in adult B cell ALL patients. J. Clin. Invest.

[8] Frigault MJ, Maus MV (2020). State of the art in CAR T cell therapy for CD19+ B cell malignancies. J. Clin. Invest.

[9] Kenderian SS (2015). CD33-specific chimeric antigen receptor T cells exhibit potent preclinical activity against human acute myeloid leukemia. Leukemia.

[10] Casucci M (2013). CD44v6-targeted T cells mediate potent antitumor effects against acute myeloid leukemia and multiple myeloma. Blood.

[11] Mardiros A (2013). T cells expressing CD123-specific chimeric antigen receptors exhibit specific cytolytic effector functions and antitumor effects against human acute myeloid leukemia. Blood.

[12] Tashiro H (2017). Treatment of acute myeloid leukemia with T cells expressing chimeric antigen receptors directed to C-type lectin-like molecule 1. Mol. Ther..

[13] Jetani H (2018). CAR T-cells targeting FLT3 have potent activity against FLT3(−)ITD(+) AML and act synergistically with the FLT3-inhibitor crenolanib. Leukemia.

[14] Kim MY (2018). Genetic inactivation of CD33 in hematopoietic stem cells to enable CAR T cell immunotherapy for acute myeloid leukemia. Cell.

[15] Ting J, Lee AS (1988). Human gene encoding the 78,000-dalton glucose-regulated protein and its pseudogene: structure, conservation, and regulation. DNA.

[16] Bertolotti A, Zhang Y, Hendershot LM, Harding HP, Ron D (2000). Dynamic interaction of BiP and ER stress transducers in the unfolded-protein response. Nat. Cell Biol..

[17] Llewellyn DH, Roderick HL, Rose S (1997). KDEL receptor expression is not coordinatedly up-regulated with ER stress-induced reticuloplasmin expression in HeLa cells. Biochem. Biophys. Res. Commun..

[18] Trychta KA, Back S, Henderson MJ, Harvey BK (2018). KDEL Receptors are differentially regulated to maintain the ER proteome under calcium deficiency. Cell Rep..

[19] Tsai YL (2018). Endoplasmic reticulum stress activates SRC, relocating chaperones to the cell surface where GRP78/CD109 blocks TGF-beta signaling. Proc. Natl Acad. Sci. USA.

[20] Tsai YL (2015). Characterization and mechanism of stress-induced translocation of 78-kilodalton glucose-regulated protein (GRP78) to the cell surface. J. Biol. Chem..

[21] Lee AS (2001). The glucose-regulated proteins: stress induction and clinical applications. Trends Biochem. Sci..

[22] Staquicini DI (2018). Therapeutic targeting of membrane-associated GRP78 in leukemia and lymphoma: preclinical efficacy in vitro and formal toxicity study of BMTP-78 in rodents and primates. Pharmacogenomics J..

[23] Kim Y (2006). Targeting heat shock proteins on cancer cells: selection, characterization, and cell-penetrating properties of a peptidic GRP78 ligand. Biochemistry.

[24] Bolouri H (2018). The molecular landscape of pediatric acute myeloid leukemia reveals recurrent structural alterations and age-specific mutational interactions. Nat. Med..

[25] Cancer Genome Atlas Research, N. (2013). Genomic and epigenomic landscapes of adult de novo acute myeloid leukemia. N. Engl. J. Med..

[26] Haferlach T (2010). Clinical utility of microarray-based gene expression profiling in the diagnosis and subclassification of leukemia: report from the International Microarray Innovations in Leukemia Study Group. J. Clin. Oncol..

[27] Bagger FO (2016). BloodSpot: a database of gene expression profiles and transcriptional programs for healthy and malignant haematopoiesis. Nucleic Acids Res..

[28] Rapin N (2014). Comparing cancer vs normal gene expression profiles identifies new disease entities and common transcriptional programs in AML patients. Blood.

[29] Brown CE (2018). Optimization of IL13Ralpha2-targeted chimeric antigen receptor T cells for improved anti-tumor efficacy against glioblastoma. Mol. Ther..

[30] Kolonin MG, Saha PK, Chan L, Pasqualini R, Arap W (2004). Reversal of obesity by targeted ablation of adipose tissue. Nat. Med.

[31] Miao YR (2013). Inhibition of established micrometastases by targeted drug delivery via cell surface-associated GRP78. Clin. Cancer Res.

[32] Epperly, R., Gottschalk, S. & Velasquez, M. P. Harnessing T cells to target pediatric acute myeloid leukemia: CARs, BiTEs, and Beyond. *Children (Basel)* **7**, 14 (2020). 10.3390/children7020014 PMC7072334 32079207

[33] Boyd AL (2018). Identification of chemotherapy-induced leukemic-regenerating cells reveals a transient vulnerability of human AML recurrence. Cancer Cell.

[34] Zhang Y (2013). Cancer cells resistant to therapy promote cell surface relocalization of GRP78 which complexes with PI3K and enhances PI(3,4,5)P3 production. PLoS ONE.

[35] Sano R, Reed JC (2013). ER stress-induced cell death mechanisms. Biochim. Biophys. Acta.

[36] Iurlaro R, Munoz-Pinedo C (2016). Cell death induced by endoplasmic reticulum stress. FEBS J..

[37] Wey S (2012). Inducible knockout of GRP78/BiP in the hematopoietic system suppresses Pten-null leukemogenesis and AKT oncogenic signaling. Blood.

[38] Ota J (2003). Proteomic analysis of hematopoietic stem cell-like fractions in leukemic disorders. Oncogene.

[39] Haubner S (2019). Coexpression profile of leukemic stem cell markers for combinatorial targeted therapy in AML. Leukemia.

[40] Zhang H (2021). Anti-CLL1 chimeric antigen receptor T-cell therapy in children with relapsed/refractory acute myeloid leukemia. Clin. Cancer Res.

[41] Perna F (2017). Integrating proteomics and transcriptomics for systematic combinatorial chimeric antigen receptor therapy of AML. Cancer Cell.

[42] Pameijer CR (2007). Conversion of a tumor-binding peptide identified by phage display to a functional chimeric T cell antigen receptor. Cancer Gene Ther..

[43] Whilding LM (2017). Targeting of aberrant alphavbeta6 integrin expression in solid tumors using chimeric antigen receptor-engineered T cells. Mol. Ther..

[44] Liu Y (2007). Mechanistic studies of a peptidic GRP78 ligand for cancer cell-specific drug delivery. Mol. Pharm..

[45] Yoneda Y (2008). A cell-penetrating peptidic GRP78 ligand for tumor cell-specific prodrug therapy. Bioorg. Med Chem. Lett..

[46] Mata M (2017). Inducible activation of MyD88 and CD40 in CAR T cells results in controllable and potent antitumor activity in preclinical solid tumor models. Cancer Discov..

[47] Shum T (2017). Constitutive signaling from an engineered IL7 receptor promotes durable tumor elimination by tumor-redirected T cells. Cancer Discov..

[48] Rafiq S, Hackett CS, Brentjens RJ (2020). Engineering strategies to overcome the current roadblocks in CAR T cell therapy. Nat. Rev. Clin. Oncol..

[49] Hu B (2017). Augmentation of antitumor immunity by human and mouse CAR T cells secreting IL-18. Cell Rep..

[50] Avanzi MP (2018). Engineered tumor-targeted T cells mediate enhanced anti-tumor efficacy both directly and through activation of the endogenous immune system. Cell Rep..

[51] Ma X (2020). Interleukin-23 engineering improves CAR T cell function in solid tumors. Nat. Biotechnol..

[52] Misra UK, Gonzalez-Gronow M, Gawdi G, Pizzo SV (2005). The role of MTJ-1 in cell surface translocation of GRP78, a receptor for alpha 2-macroglobulin-dependent signaling. J. Immunol..

[53] Araujo N, Hebbar N, Rangnekar VM (2018). GRP78 is a targetable receptor on cancer and stromal cells. EBioMedicine.

[54] Kawalekar OU (2016). Distinct signaling of coreceptors regulates specific metabolism pathways and impacts memory development in CAR T cells. Immunity.

[55] Mestermann, K. et al. The tyrosine kinase inhibitor dasatinib acts as a pharmacologic on/off switch for CAR T cells. *Sci Transl. Med*. **11**, eaau5907 (2019). 10.1126/scitranslmed.aau5907 PMC7523030 31270272

[56] Weber EW (2019). Pharmacologic control of CAR-T cell function using dasatinib. Blood Adv..

[57] O’Hare T (2005). Combined Abl inhibitor therapy for minimizing drug resistance in chronic myeloid leukemia: Src/Abl inhibitors are compatible with imatinib. Clin. Cancer Res.

[58] Shah NP (2004). Overriding imatinib resistance with a novel ABL kinase inhibitor. Science.

[59] Rasche L (2015). GRP78-directed immunotherapy in relapsed or refractory multiple myeloma - results from a phase 1 trial with the monoclonal immunoglobulin M antibody PAT-SM6. Haematologica.

[60] Klco JM (2014). Functional heterogeneity of genetically defined subclones in acute myeloid leukemia. Cancer Cell.

[61] Bonifant CL (2016). CD123-Engager T cells as a novel immunotherapeutic for acute myeloid leukemia. Mol. Ther.

[62] Faber ZJ (2016). The genomic landscape of core-binding factor acute myeloid leukemias. Nat. Genet..

[63] Anders S, Pyl PT, Huber W (2015). HTSeq-a Python framework to work with high-throughput sequencing data. Bioinformatics.

[64] Leek JT, Storey JD (2007). Capturing heterogeneity in gene expression studies by surrogate variable analysis. PLoS Genet.

[65] Ritchie ME (2015). limma powers differential expression analyses for RNA-sequencing and microarray studies. Nucleic Acids Res..

[66] Ahmed N (2009). Immunotherapy for osteosarcoma: genetic modification of T cells overcomes low levels of tumor antigen expression. Mol. Ther..

[67] Diaconu I (2017). Inducible caspase-9 selectively modulates the toxicities of CD19-specific chimeric antigen receptor-modified T cells. Mol. Ther..

