# Supplementary material for: CAR T cells redirected to cell surface GRP78 display robust anti-acute myeloid leukemia activity and do not target hematopoietic progenitor cells
Source: Nat Commun. 2022 Jan 31;13:587. doi: 10.1038/s41467-022-28243-6 (PMC8803836; doi:10.1038/s41467-022-28243-6)
Supplement: Supplementary file 1 — Supplementary Information [file 41467_2022_28243_MOESM1_ESM.pdf]

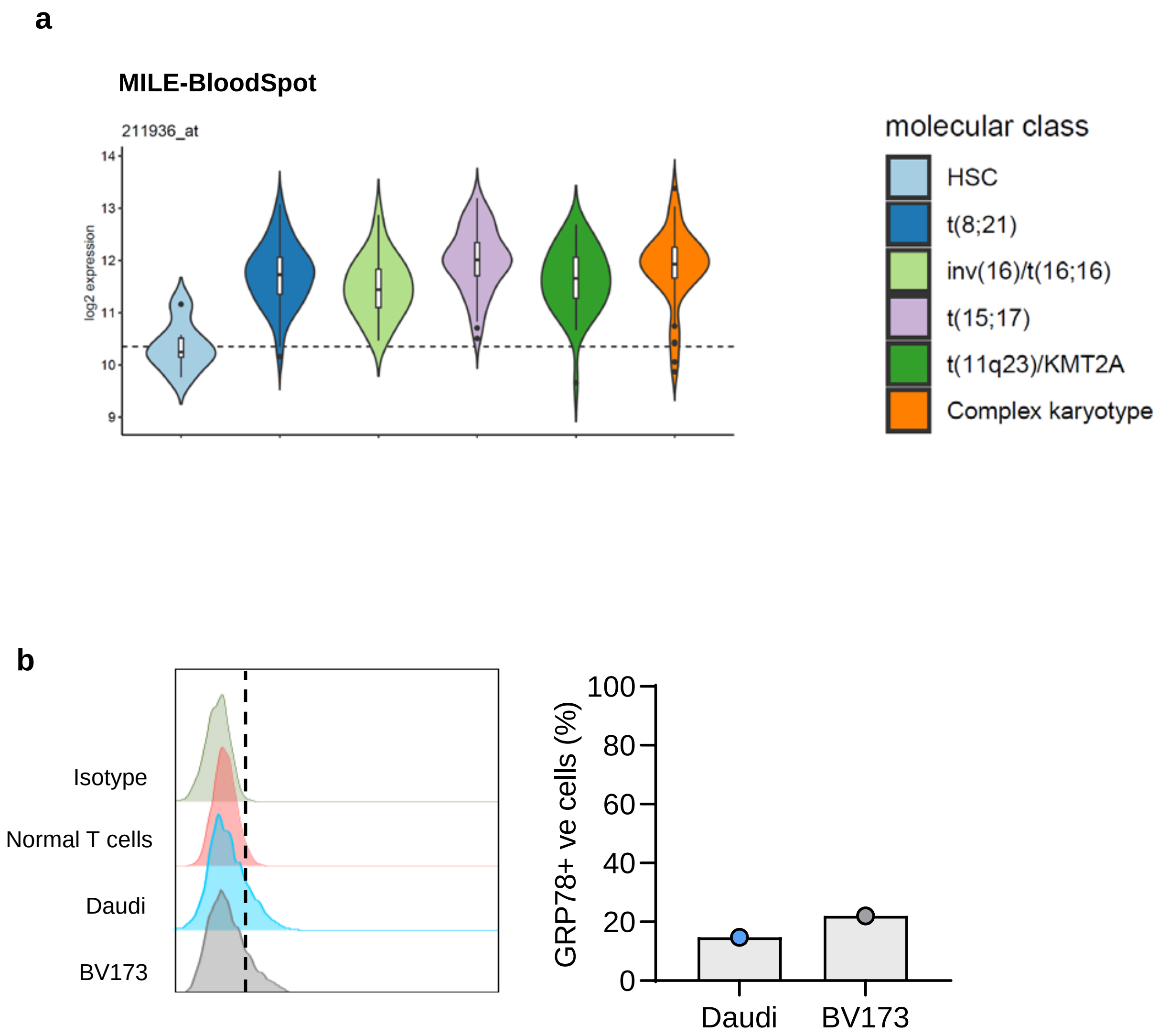

Supplementary Figure 1: a. GRP78 gene expression Violin plots of GRP78 (HSPA5) gene expression by microarray analysis of AML samples present in the MILE study as compared to normal HSCs (N=252, HSCs vs AML; T-test with pairwise comparisons, p<0.0001). The properties of the box-plots are defined as follows; minima: lower whisker = smallest observation greater than or equal to lower hinge - 1.5*IQR (IQR=interquartile range: the difference between the 75th and 25th percentiles), box lower hinge = 25% quantile, box middle = median, 50% quantile, box upper hinge = 75% quantile, maxima: upper whisker = largest observation less than or equal to upper hinge + 1.5*IQR. **b. GRP78 surface expression on lymphoid tumors.** (Right panel) Flow cytometric analysis showing GRP78 surface expression on normal T cells and lymphoid tumors Daudi and BV173, (left panel) Graph showing percentage of GRP78+ve cells.

a

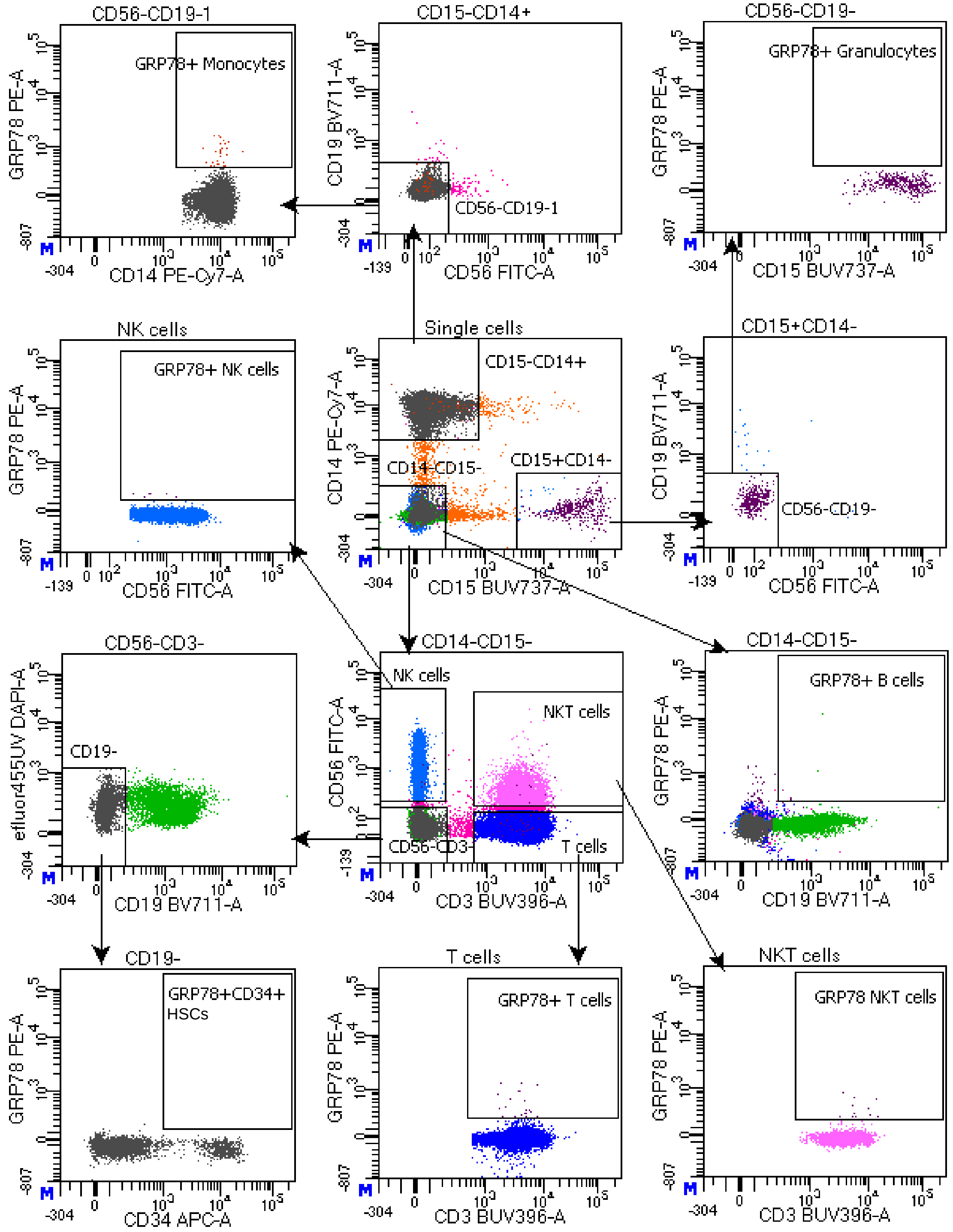

Supplementary Figure 2a: GRP78 expression on myeloid and lymphoid lineages from peripheral blood. a. Peripheral blood was collected from healthy donors (n=4). Following RBC lysis, single cell suspensions were stained for GRP78, hCD3, hCD14, hCD15, hCD19, hCD34, hCD56 and analyzed by flow cytometry to determine the presence of cell surface GRP78 on different cell subtypes**;** representative dot plots shown. The lineages were defined as follows; Monocytes: CD15-/CD14+; Granulocytes:CD15+/CD14-; NK cells: CD56+/CD3- ; HSCs: CD34+/CD19-/CD56-/CD3- ; T cells: CD3+/CD56-; NKT cells: CD56+/CD3+ ; B-cells: CD19+/CD14-/CD15-.

Supplementary Figure. 2a

b

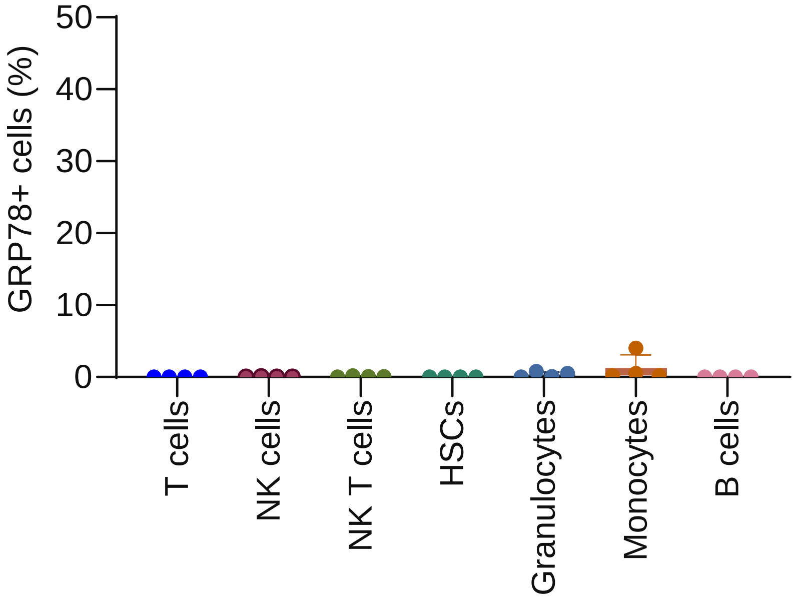

Supplementary Figure 2b: GRP78 expression on myeloid and lymphoid lineages from peripheral blood. b. Graph showing the percentage of cells expressing GRP78 at the cell surface.

a

Primary AML	Diagnosis	Recurrent Molecular Findings	Source (BM/PB)
AML 1	AML, FAB, M2	None identified	BM
AML 9	AML (FAB M1)	N.D.	BM
AML 2	AML (FAB M4)	NUP98-NSD1	PB
AML 3	AML (FAB M5)	NUP98-NSD1	PB
AML 11	AML (FAB M4)	NUP98-NSD1	BM
AML 14	AML (FAB M5)	NPM1c	BM
AML 8	AML, relapse	NUP98 NSD1	BM
AML10	AML, relapse	KMT2A-MLLT4	BM
AML12	AML, relapse	KAT6A-CREBBP	BM
AML 13	AML, relapse	KMT2A-MLLT3	BM
AML 6	Therapy-related AML	KMT2A-MLLT3	BM
AML 5	Therapy-related AML	KMT2A-MLLT3	BM
AML 4	Therapy-related AML	KMT2A-MLLT3, FLT3-ITD	PB
AML 7	Therapy-related AML	KMT2A-MLLT3	BM

b

AML-PDX	ID	Diagnosis	Molecular Findings	Source (BM/PB)
AML-PDX 1	FWV75	AML with myelodysplasia related changes	DEK-NUP214	BM>PDX
AML-PDX 2	GWK6C	AML (without maturation, FAB M2)	NPM1-MLF1, FLT3 ITD	BM>PDX
AML-PDX 3	8KG5	Therapy-related AML	KMT2A-MLLT10	BM>PDX
AML-PDX 4	NPxP4	Therapy-related AML, relapse	KMT2A-MLLT3	BM>PDX
AML-PDX 5	GQHGJ	MPAL, relapse	CBL, EZH2, TP53, ETV6	BM>PDX

Supplementary Table 1: AML sample description. Description of **a.** primary AML and **b.** AML-PDX samples used for the studies shown in Figures 1 and 2.
BM-Bone Marrow; PB- Peripheral Blood; N.D.- Not Determined

a

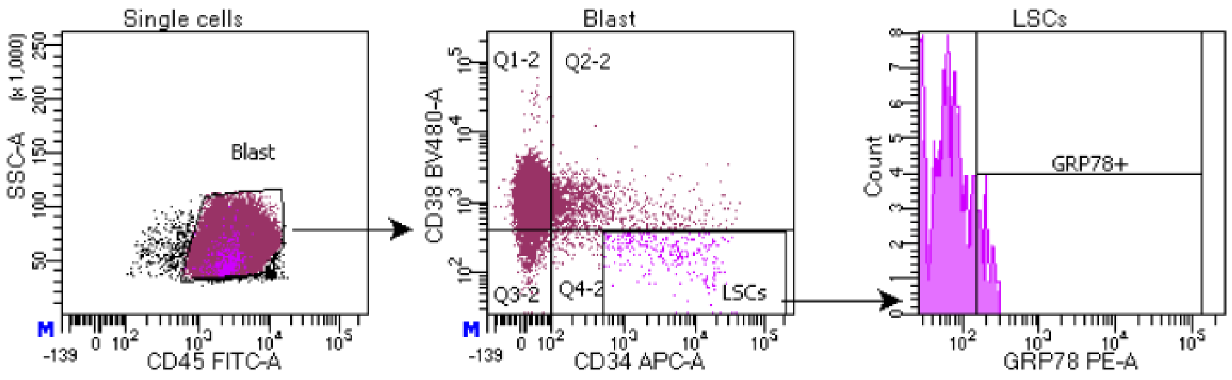

b

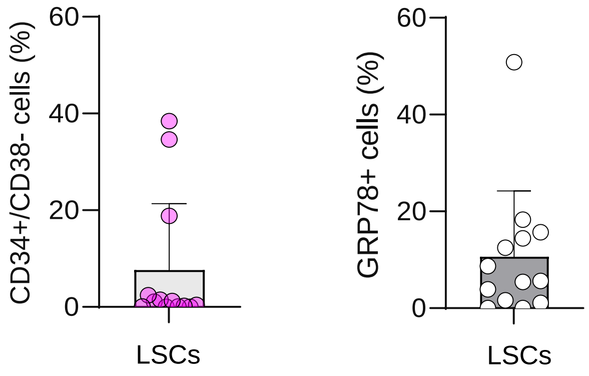

Supplementary Figure 3: GRP78 expression on LSCs. a. Primary AML samples (n=13) were stained for GRP78, hCD38, hCD34, hCD45 and analyzed by flow cytometry to determine the presence of cell surface GRP78 on LSCs (CD34+/CD38-)**;** representative dot plots shown. **b.** Graph showing the percentage of LSCs expressing cell surface GRP78.

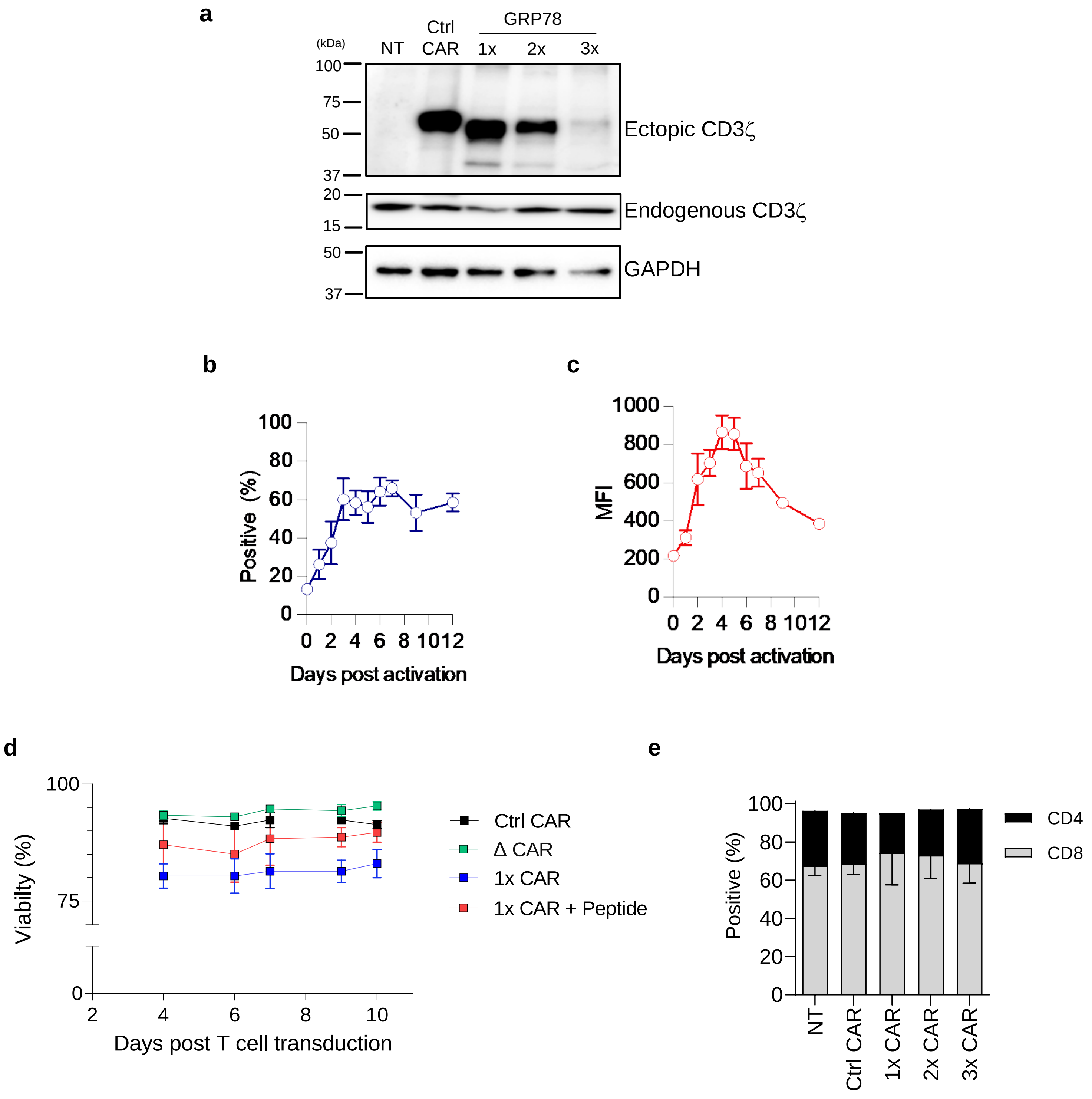

Supplementary Figure 4: CAR expression, transduction in the presence of free GRP78-specific peptide, and immunophenotype of GRP78-CAR T cells. a. Western blot analysis of T cells transduced with a control CAR (Ctrl CAR), GRP78.1x-CAR (1x CAR), GRP78.2x-CAR (2x CAR) or GRP78.3x-CAR (3x CAR). N=3, representative blot shown. **b.** and **c.** T cells were activated using CD3 and CD28 antibodies. Cell surface expression was determined by flow cytometry. **b.** Percent of GRP78+ cells. **c.** mean fluorescent intensity (MFI). (Days 0,1,2,4,5,7,9,12: N=3; Days 3,6: N=6). Mean +/-SEM is shown. **d.** Cell viability measured by trypan blue staining from day 4 to 10. GRP78.1x-CAR T cells were cultured +/- GRP78 peptide (3μM), N=3 **e.** Flow cytometric analysis of CD4:CD8 ratio for NT and CAR T cells. N=3..

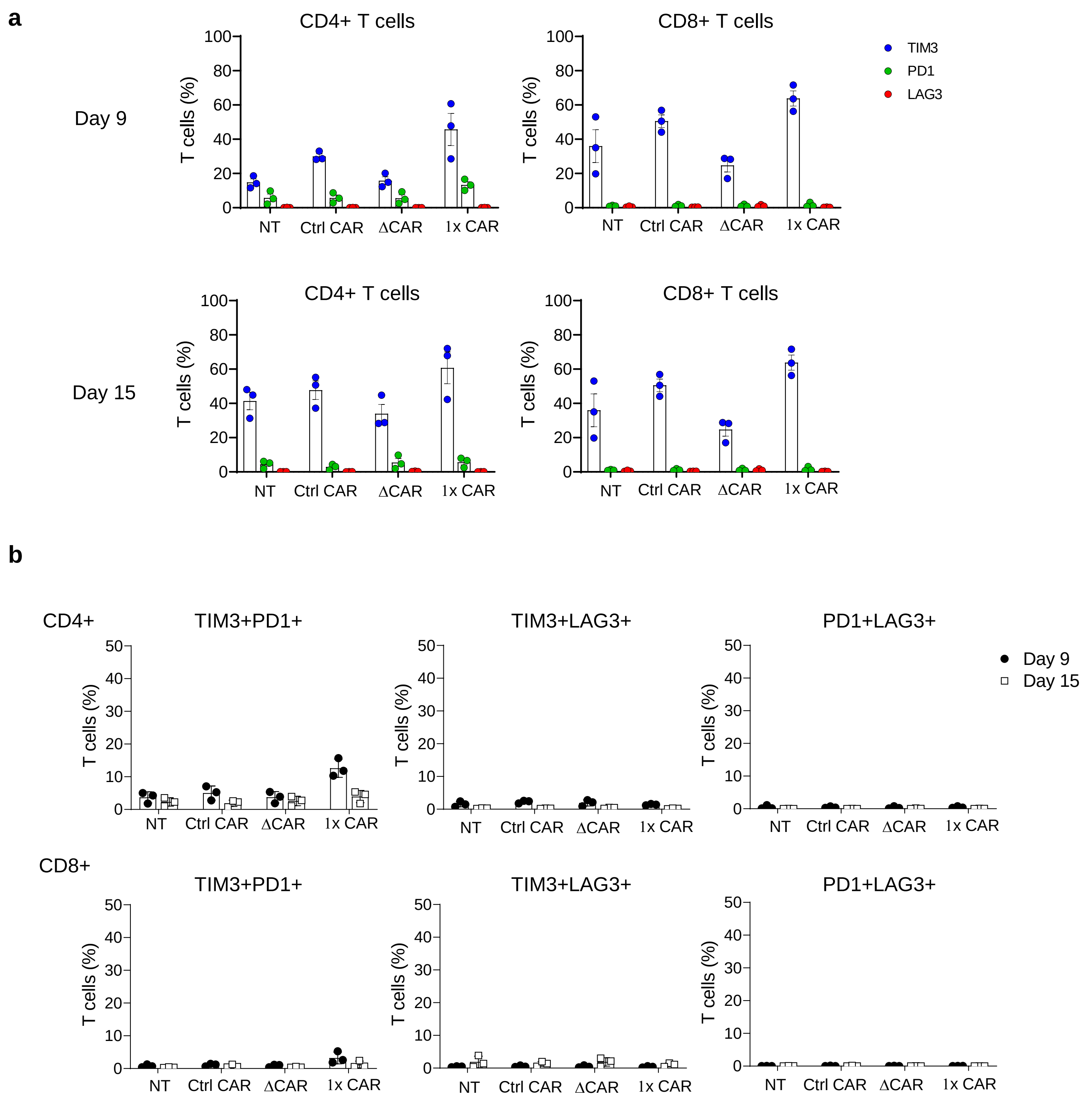

Supplementary Figure 5: TIM3, PD-1, LAG3 expression on GRP78-CAR T cells. Non-transduced NT, control CAR, ΔCAR and GRP78 1x CAR T cells were stained for hCD4, hCD8, TIM3, PD-1 and LAG3 and analyzed by flow cytometry on day 9 and day 15 post transduction. **a.** Graphs showing percentage of TIM3, PD-1 and LAG3 expression on CD4+ and CD8+ T cells. **b.** Graphs showing percentage of double positive T cells (TIM3+PD1+, TIM3+LAG3+, PD1+LAG3+). N= 3. Error bars denote means +/-SEM

a

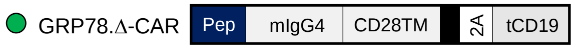

b

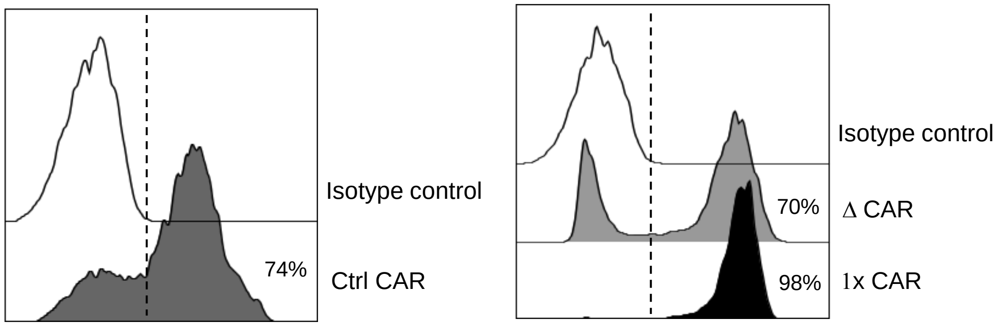

c

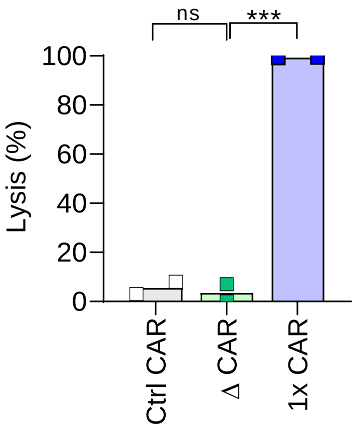

Supplementary Figure 6: Expression and activity of GRP78.Δ-CAR. a. Scheme of retroviral vector encoding GRP78.Δ-CAR. **b.** Flow cytometric analysis of GRP78.Δ-CAR expression. Control CAR- and NT T cells were used for isotype controls. **c.** MOLM13 cells were cocultured for 24 hours with a control CAR (Ctrl CAR), GRP78 1x peptide CAR (1x CAR) or GRP78.Δ-CAR (Δ CAR). Cytotoxicity was measured using a luciferase cytotoxicity assay. N=2. ***p=0.0002. Ordinary one-way ANOVA was used.

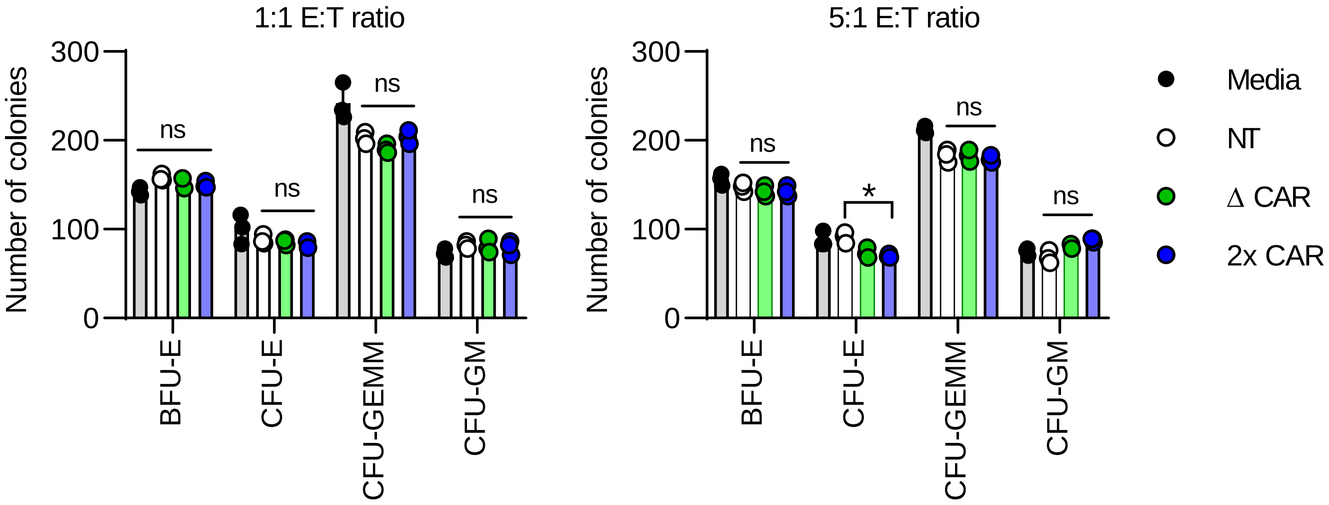

Supplementary Figure 7: GRP78-CAR T cells do not recognize hematopoietic progenitor cells (HPCs). Indicated effector T cells were incubated with HPCs for 4 h at E:T ratios of 1:1 and 5:1, plated on semisolid media, and BFU-E (Burst Forming Unit – erythroid) and CFU colonies (Colony-forming unit – erythroid: CFU-E, Colony-forming unit − granulocyte, erythroid, macrophage, megakaryocyte: CFU-GEMM), were enumerated after 12–14 days; N=3, technical replicates; one-way ANOVA and T-test for pairwise comparison; *p=0.0109; ns; not significant.

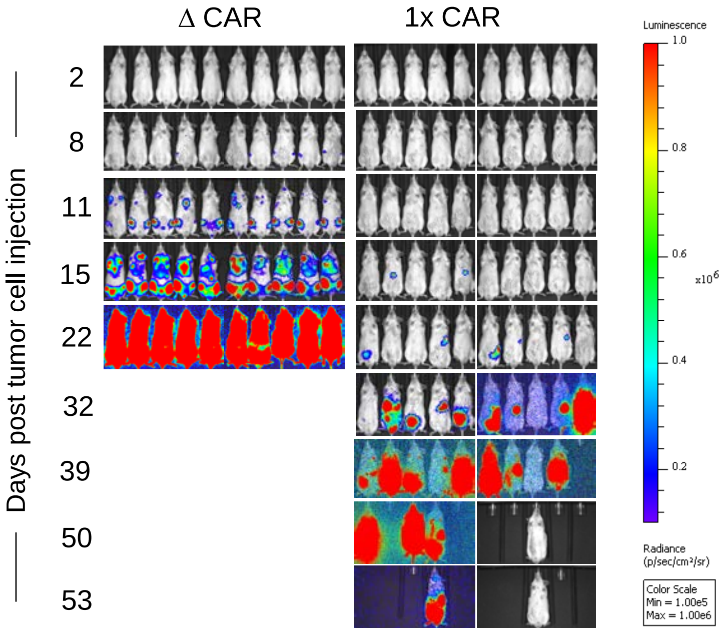

Supplementary Figure 8: GRP78-CAR T cells have antitumor activity against MOLM13. Supplementary figure showing the IVIS images for the experiment shown in **Fig. 6**.

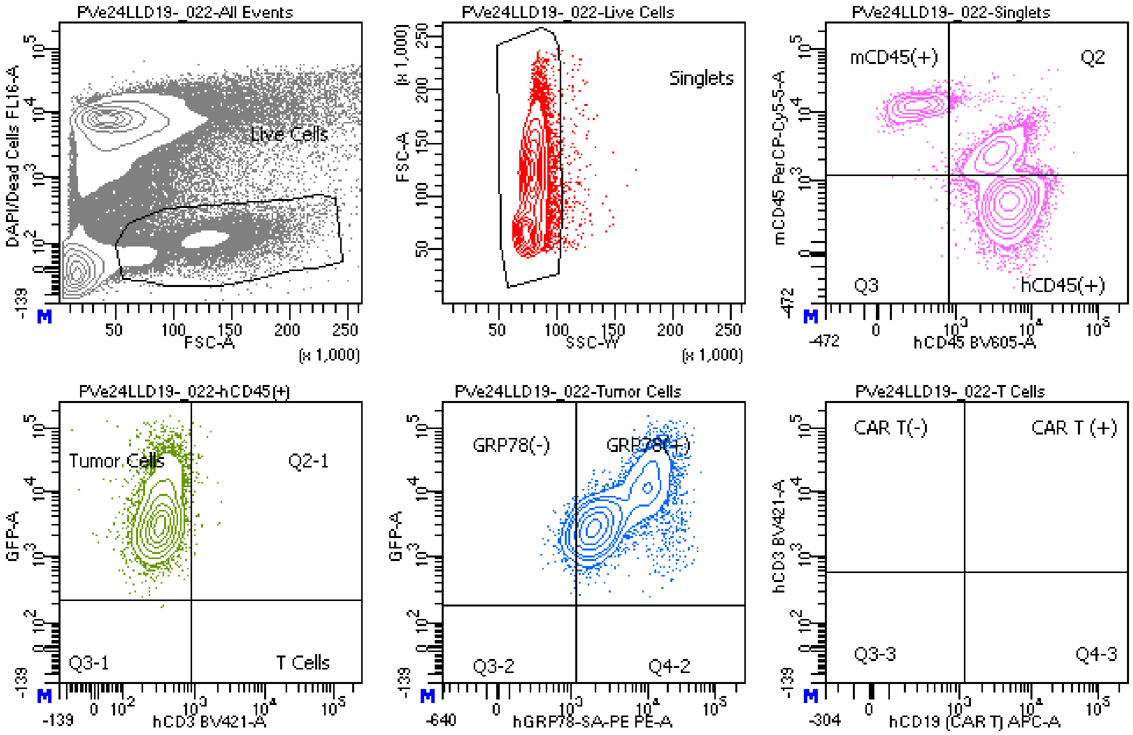

Supplementary Figure 9: GRP78 expression remains intact on recurrent tumors. Supplementary figure showing gating strategy for GRP78 staining for the experiment shown in **Fig. 6**.

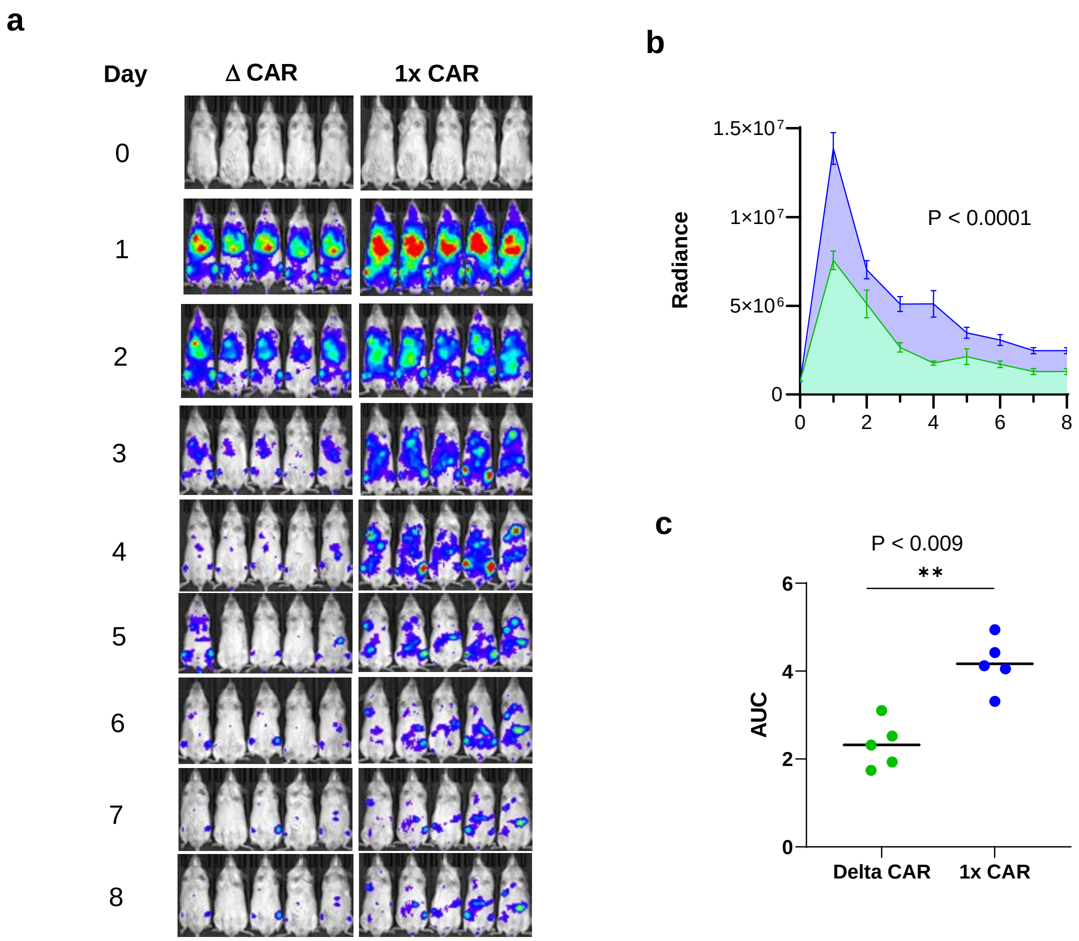

Supplementary Figure 10: In vivo persistence of GRP78 CAR T cells. MOLM13 xenograft model. NSG mice were injected with MOLM13 ($5x10^3$) cells i.v. (tail vein), and on day 7 received a single i.v. dose of $3x10^6$ T cells co-transduced with GFP-ffluc. IVIS imaging was performed daily. **a**. Bioluminescence data (total flux=photons/sec) . **b** Graph showing total flux (N=5, Two-way ANOVA, p<0.0001) **c.** Area under the curve analysis (AUC, **p<0.009)

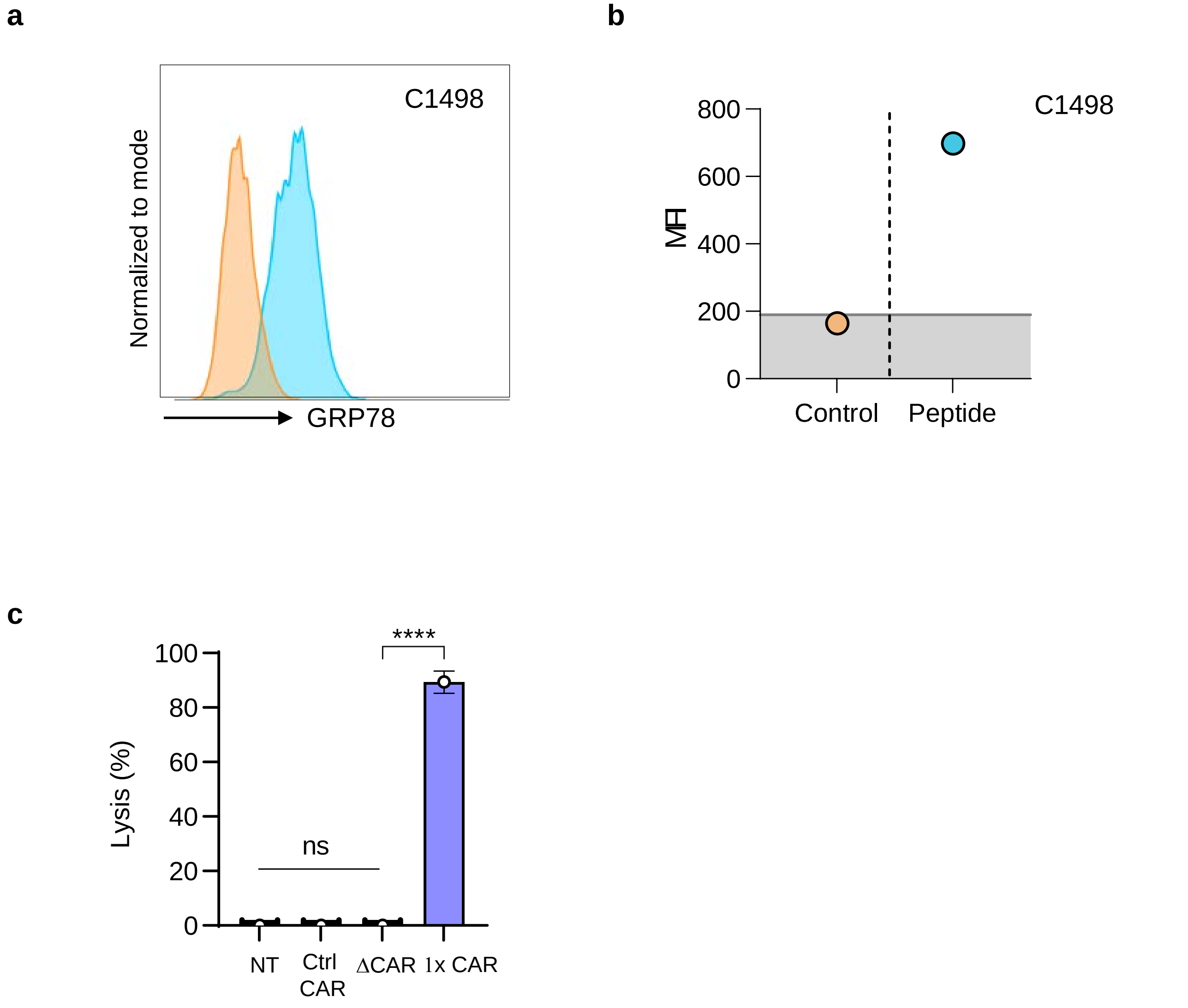

Supplementary Figure Fig. 11: GRP78 is expressed on the surface of murine tumors A. C1498 murine AML cells were stained with GRP78 peptide (Biotin-Ahx-CTVALPGGYVRVC) and analyzed by Flow cytometry. **B.** Graph showing expression of GRP78 on C1498 **C.** Graph showing % Lysis of C1498 murine AML cells. C1498 target cells were cocultured for 24 hours with human effector T cells (NT, Control, Delta or GRP78 1x-CAR T cells) at an E:T ratio of 2:1. N=3; One way ANOVA, ****p<0.0001. ns, not significant.

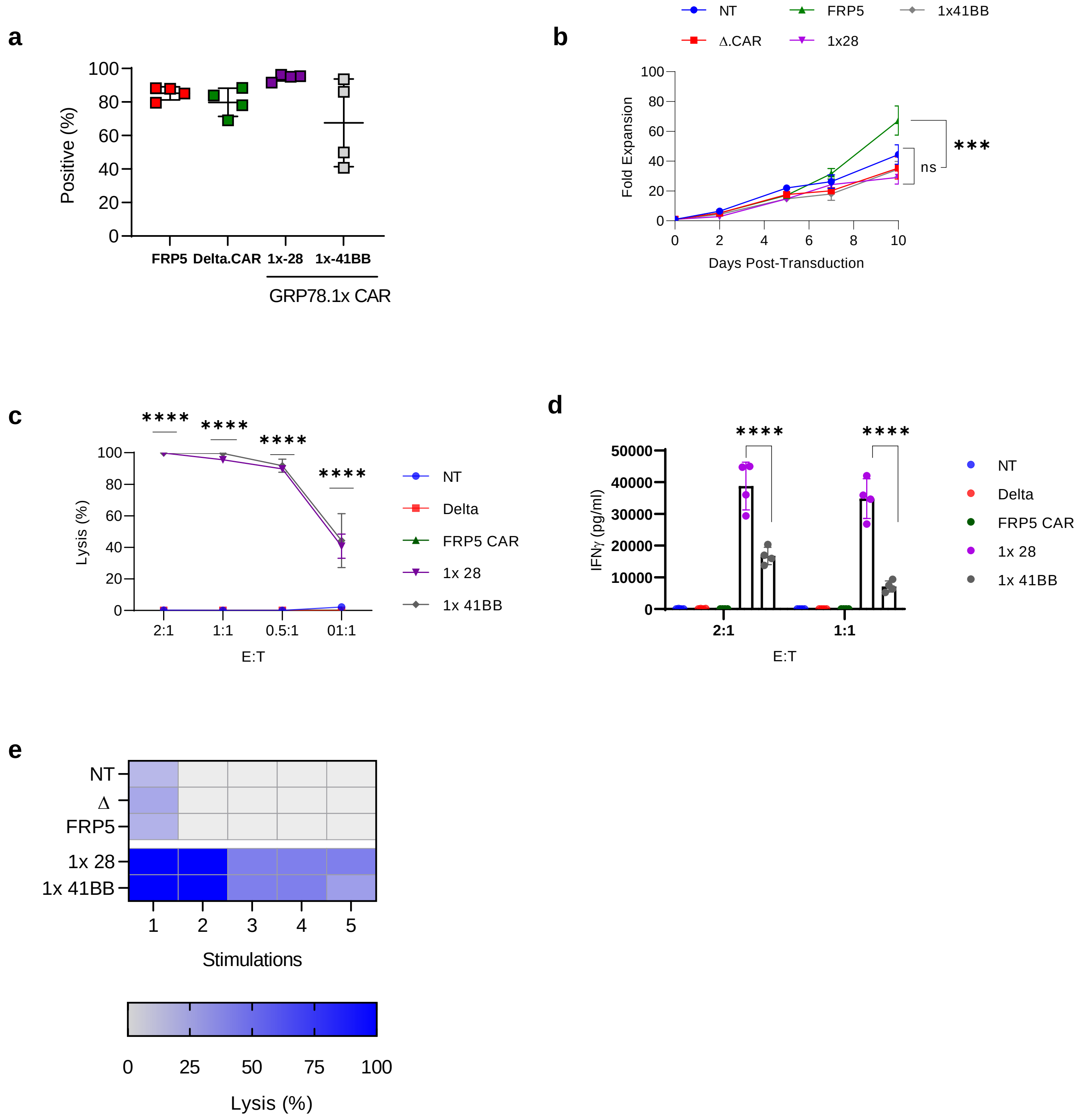

Supplementary Figure 12: In vitro comparison of GRP78-CAR with either a CD28 or a 41BB costimulation domain. a. Transduction efficiency of controls (FRP5 and Delta.CAR) as well as of 1x-28 and 1x-41BB **b.** Expansion of constructs measured up o 10 days post transduction (N=4, Two-way ANOVA with multiple comparisons, ***p<0.001. c. Tumor lysis as evaluated by luciferase-based analysis. N=4, Two-way ANOVA with multiple comparisons ****p<0.0001. d. IFN-g ELISA at 2:1 and 1:1 (N=4, Two-way ANOVA with multiple comparisons ****p<0.0001) **e.** Serial stimulation assay (N=4)

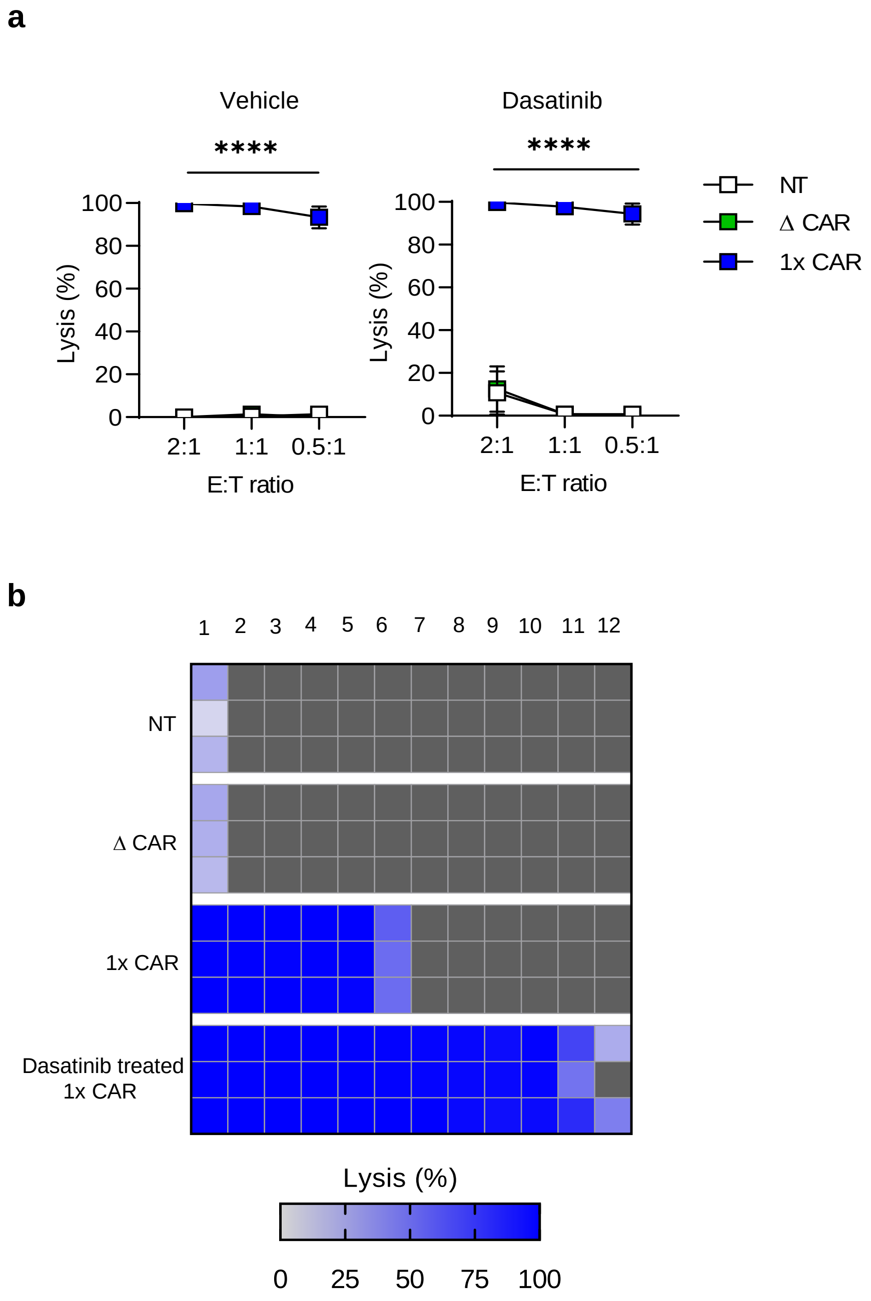

Supplementary Figure 13: Dasatinib improves persistence of GRP78 CAR T cells in vitro. a. Graph showing cytolysis of MOLM13 AML cells. MOLM13 target cells were cocultured with human effector T cells (NT, Delta or GRP78 1x-CAR T cells) for 24 hours at three E:T ratios (2:1, 1:1, 0.5:1). N=3; Two-way ANOVA, **** p<0.0001. **b.** Heat map showing cytolysis of MOLM13 AML cells in a serial stimulation assay. Fresh MOLM13 cells were added every 72 hours. N=3.
